# Perturbations in gut microbiota composition in patients with autoimmune neurological diseases: a systematic review and meta-analysis

**DOI:** 10.3389/fimmu.2025.1513599

**Published:** 2025-02-06

**Authors:** Xiaolin Deng, Xue Gong, Dong Zhou, Zhen Hong

**Affiliations:** ^1^ Department of Neurology, West China Hospital, Sichuan University, Chengdu, Sichuan, China; ^2^ Department of Neurology, Chengdu Shangjin Nanfu Hospital, Chengdu, Sichuan, China

**Keywords:** gut microbiota, gut dysbiosis, autoimmune neurological diseases, systematic review, meta-analysis

## Abstract

Studies suggest that gut dysbiosis occurs in autoimmune neurological diseases, but a comprehensive synthesis of the evidence is lacking. Our aim was to systematically review and meta-analyze the correlation between the gut microbiota and autoimmune neurological disorders to inform clinical diagnosis and therapeutic intervention. We searched the databases of PubMed, Embase, Web of Science, and the Cochrane Library until 1 March 2024 for research on the correlation between gut microbiota and autoimmune neurological disorders. A total of 62 studies provided data and were included in the analysis (*n* = 3,126 patients, *n* = 2,843 healthy individuals). Among the included studies, 42 studies provided data on α-diversity. Regarding α-diversity, except for Chao1, which showed a consistent small decrease (SMD = −0.26, 95% CI = −0.45 to −0.07, *p* < 0.01), other indices demonstrated no significant changes. While most studies reported significant differences in β-diversity, consistent differences were only observed in neuromyelitis optica spectrum disorders. A decrease in short-chain fatty acid (SCFA)-producing bacteria, including *Faecalibacterium* and *Roseburia*, was observed in individuals with autoimmune encephalitis, neuromyelitis optica spectrum disorders, myasthenia gravis, and multiple sclerosis. Conversely, an increase in pathogenic or opportunistic pathogens, including *Streptococcus* and *Escherichia-Shigella*, was observed in these patients. Subgroup analyses assessed the confounding effects of geography and immunotherapy use. These findings suggest that disturbances of the gut flora are associated with autoimmune neurological diseases, primarily manifesting as non-specific and shared microbial alterations, including a reduction in SCFA-producing bacteria and an increase in pathogenic or opportunistic pathogens.

**Systematic review registration:**

https://www.crd.york.ac.uk/prospero/, identifier CRD42023410215.

## 1 Introduction

The intestinal microecological community is intimately associated with human neurodevelopment, and disruptions in gut flora are increasingly recognized as contributors to various diseases through immunomodulatory and metabolic pathways. Both preclinical and clinical research have emphasized the importance of the gut flora dysbiosis in conditions like depression, rheumatoid arthritis, and multiple sclerosis (MS) (1–3). Growing evidence suggests that disruptions in composition of gut microbiota and their immunomodulatory functions are implicated in various autoimmune neurological diseases, including MS, optic neuromyelitis optica spectrum disorders (NMOSD), myasthenia gravis (MG), and autoimmune encephalitis (AIE) (4–7). Moreover, recent research has demonstrated that interventions such as fecal transplantation or probiotic supplementation hold promise in mitigating disease progression (4, 8–11). Therefore, regulating gut microbiota homeostasis has the potential to serve as a diagnostic tool or therapeutic approach.

However, current research on gut microbiota in autoimmune neurological diseases yields varied findings, lacking a comprehensive synthesis. There is consensus that elevated α-diversity indicates a healthy gut microecology. However, diseases such as AIE, MS, and MG showed different patterns in gut microbiota α-diversity. Some studies reported reduced α-diversity, while others showed no significant change (6, 12–14). This variability in findings suggests that the relationship between gut flora α-diversity and autoimmune neurological disorders is complex and may be influenced by various factors, including measurement methods, environmental influences, and drug use. Moreover, identifying specific alterations in gut microbiota, whether increased or decreased, holds promise for enhancing disease diagnostic accuracy, deepening disease understanding, and informing treatment strategies. Identifying non-specific and common gastrointestinal microbes in different autoimmune neurological diseases is also important. Such insights could enhance our understanding of the common pathogenesis underlying these disorders. Hence, distinguishing between specific and shared gut microbial changes across a broader spectrum of autoimmune neurological diseases is imperative.

To date, no comprehensive investigation has systematically examined changes in gut microbiota across a spectrum of autoimmune neurological diseases. To address this gap, we performed an updated systematic review and meta-analysis to integrate mounting evidence regarding the connection between gut microbiota and autoimmune neurological disorders.

## 2 Method

Pre-registration of this meta-analysis was undertaken through PROSPERO (CRD42023410215). We followed the reporting guidelines of Preferred Reporting Items for Systematic Reviews and Meta-Analyses (PRISMA), along with the Cochrane Guidelines for Comprehensive and Updated Reviews (15, 16).

### 2.1 Search strategy

We performed a comprehensive literature search of the PubMed, Cochrane, Embase, and Web of Science databases up to 1 March 2024. Autoimmune neurological diseases involve the immune system’s erroneous assault on the nervous system, sparking inflammation and neural damage. These conditions can affect the brain, spinal cord, and peripheral nerves, resulting in various symptoms depending on the affected region (17). The study focused on a range of such conditions, including MS, AIE, MG, NMOSD, neuro-Behcet’s disease (NBD), acute disseminated encephalomyelitis, and Guillain–Barré syndrome. Comprehensive search details are provided in **Supplementary Table S1**.

### 2.2 Selection criteria

Two independent investigators, XL Deng and X Gong, used Endnote to screen studies for inclusion in this evaluation. Initially, studies were screened by title and abstract to exclude those not meeting the eligibility criteria, followed by a full-text review for further evaluation. Any discrepancies between investigators regarding study inclusion were discussed to establish consensus. We included studies that fulfilled the following criteria: (1) implementation of observational designs including case–control studies, cross-sectional studies, cohort studies, or baseline data of intervention studies; (2) analysis of gut microbiota using available diversity or abundance data; (3) inclusion of populations diagnosed with autoimmune neurological diseases; and (4) publication of full-text articles in English in peer-reviewed journals. In cases where data were duplicated or shared between studies, the most recent and comprehensive dataset was used for analysis.

### 2.3 Data extraction

XL Deng and X Gong extracted data from full-text articles using a pre-designed template, which was then cross-checked for accuracy. The primary outcomes and metrics were α-diversity, β-diversity, and the relative abundance of intestinal microbes. α-diversity provides an overview of the microbial community within a single sample and is used to compare across different groups assessing richness (number of taxa) and evenness (the degree to which each taxon is represented). β-diversity serves as a metric for assessing the diversity among samples and examining the phylogenetic composition of communities relative to other analyzed samples (1, 18). Additionally, other pertinent information such as publication details, participant demographics, and methodologies was extracted.

### 2.4 Quality assessment

The quality of the included research was assessed using the Newcastle–Ottawa Quality Assessment Scale (NOS) (19). Scores of 5 or lower were considered low quality, scores of 6 or 7 signified moderate quality, and scores of 8 or 9 indicated high quality. XL Deng and X Gong independently conducted assessments to determine the quality of the research involved in the analysis. In cases of score disagreement, the two reviewers collaboratively re-evaluated the articles to reach a consensus (2).

### 2.5 Quantitative synthesis

A meta-analysis was conducted to compare the α-diversity between individuals with autoimmune neurological diseases and healthy controls. The analysis included indices containing data from at least 10 research studies. The pooled standardized mean difference (SMD) and related 95% confidence interval (CI) were computed via inverse-variance random-effects meta-analysis for each index. The effect sizes were classified based on magnitude: trivial (SMD ≤ 0.2), small (0.2 < SMD < 0.5), moderate (0.5 ≤ SMD < 0.8), or large (SMD ≥ 0.8). Means and standard deviations were derived from medians and interquartile ranges. If numerical data were only presented graphically, they were extracted using WebPlotDigitizer V.4.6 (2). The DerSimonian-Laird estimator was utilized to assess heterogeneity, with significant heterogeneity defined as *I* ^2^ > 50%. Publication bias was assessed using funnel plots and the Egger’s test.

Subgroup analysis was conducted to investigate the heterogeneity among studies, examining various factors such as the type of autoimmune neurological diseases, the geographical distribution of study populations (comparing countries in the East and West), and the use of immunotherapy (including immunosuppressants, immunomodulators, and immune-reconstitution therapy), distinguishing between patients receiving treatment and those without it. Eastern countries comprise East and South Asian nations, while Western countries encompass North America, Europe, and the Middle East. Studies where at least 80% of patients received immunotherapy were classified as involving treated patients. To assess the reliability of the results, two sensitivity analyses were carried out. Studies rated as low quality (with NOS scores of 5 or less) and those without matching characteristics (including age, sex, and body mass index) were excluded due to their susceptibility to confounding bias (1, 2). All analyses were conducted using the STATA/MP 17.0, with statistical significance defined as *p*-values less than 0.05.

### 2.6 Qualitative synthesis

When significant variations in β-diversity between patients with autoimmune neurological diseases and healthy individuals were observed across all studies focusing on a particular condition, these differences were considered consistent. A qualitative synthesis was undertaken due to the extensive findings and low overlap in the relative abundance of microbial species. Intra-diagnostic and inter-diagnostic comparisons were undertaken to delineate disease-specific and common alterations. Disease-related findings reported by at least two studies per taxon were summarized, with taxa categorized as exhibiting increased, decreased, or inconsistent patterns. Inconsistency was defined as less than 75% concordance between studies. Concordant findings from two studies were deemed notable for future validation, while those from three or more studies conducted by at least two independent research teams were deemed possibly relevant to the disease. A taxon exhibiting consistent change in only one disease was identified as a potential disorder-specific response, whereas changes observed in at least three disorders were categorized as common changes (2).

## 3 Results

### 3.1 Search results

**Figure 1:**
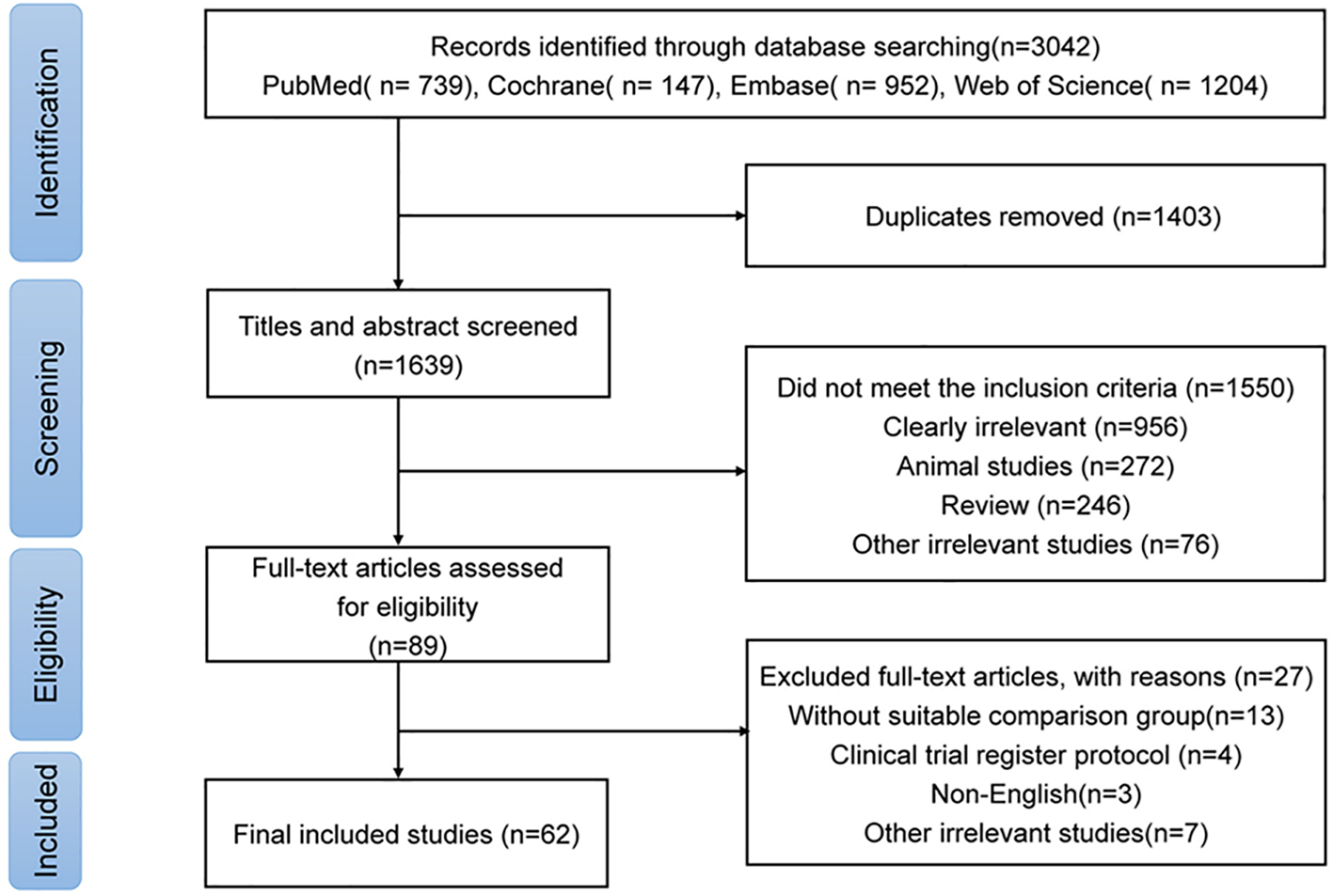
PRISMA flow diagram for studies of gut microbiome in autoimmune neurological diseases.

Initially, a total of 3,042 studies were identified (**Figure 1**). Following the removal of 1,403 duplicates, 1,550 studies that failed to fulfill the inclusion criteria were screened based on their titles and abstracts. Subsequently, 89 studies underwent full-text review, of which 27 were excluded (**Supplementary Table S2**). Finally, 62 studies focusing on six autoimmune neurological disorders (including AIE, MG, NMOSD, MS, and DON) were included (4–7, 9, 11–13, 20–73). MS was the most extensively studied disease, followed by MG, NMOSD, and AIE (**Supplementary Table S3**). The total number of participants was 3,126, ranging from 13 for NBD to 2,393 for MS. The mean age of participants varied from 32.93 years for AIE to 42.10 years for NBD (**Table 1**).

**Table 1:** Summary characteristics of the included studies.

Disorder	No.^1^			Region of studies	Mean age, years		Female ratio, %	
	Studies	Patients	Control		Patient	Control	Patient	Control
AIE	6	176	174	East: *n* = 5; West: *n* = 1	32.93	33.70	57.39	58.05
MG	7	255	213	East: *n* = 5; West: *n* = 2	41.26	38.82	54.20	51.23
NMOSD	7	235	244	East: *n* = 6; West: *n* = 1	39.22	37.85	81.70	50.82
MS	44	2,393	2,157	East: *n* = 5; West: *n* = 37; Africa: *n* = 2	41.86	41.33	69.64	53.23
NBD	1	13	14	East: *n* = 0; West: *n* = 1	42.10	37.80	38.46	28.57
DON	1	54	41	East: *n* = 1; West: *n* = 0	38.70	38.56	59.26	65.85
Total	62	3,126	2,843	East: *n* = 21; West: *n* = 39; Africa: *n* = 2	NA	NA	NA	NA

AIE, autoimmune encephalitis; MG, myasthenia gravis; NMOSD, optic neuromyelitis optica spectrum disorders; MS, multiple sclerosis; NBD, neuro-Behcet’s disease; DON, demyelinating optic neuritis; NA, not applicable; ^1^ Some include >1 disorder.

### 3.2 Study characteristics

More than half of the studies [39 (62.90%)] were conducted in Western countries (e.g., USA, Germany, UK, Italy, and Israel), 21 (33.87%) were conducted in Eastern countries (China and Japan), and only 2 (3.23%) were conducted in Africa (Egypt). Most studies on MS were conducted in Western countries [37 (84.09%)], while the majority of studies on other diseases were conducted in Eastern countries (**Supplementary Table S3**). Sample sizes for most studies were small to moderate, ranging from 7 to 500 participants. Although the exclusion criteria were generally consistent, the use of immunotherapy varied widely. Out of the studies analyzed, 22 studies (35.48%) were conducted without immunotherapy. In contrast, six studies (9.68%) included only patients who had received immunotherapy. The remaining studies did not control for this, resulting in a wide variation in the proportion of patients on immunotherapy, ranging from 9% to 95.45%. Stool processing methods and compositional analyses also varied significantly. Sequencing of 16S ribosomal RNA (16S rRNA) was the most widely applied method, used in 54 studies, followed by shotgun metagenomics in 12 studies (5 of which used metagenomics exclusively), real-time quantitative polymerase chain reaction in 2 studies, and fluorescence *in situ* hybridization in 1 study (**Supplementary Table S5**). Based on the NOS score, two studies were rated as high quality, 43 studies were rated as moderate quality, and 17 studies were rated as low quality (**Supplementary Table S4**).

### 3.3 Synthesis of α-diversity

Data on α-diversity were contributed by 42 studies, encompassing metrics of richness [observed species, abundance-based coverage estimator (ACE), Chao1, richness, and Good’s coverage), evenness (Shannon index, Simpson index, Pielou’s evenness, and evenness), and biodiversity (Faith’s phylogenetic diversity). The most frequently used measures were the observed species, ACE, Chao1, Shannon index, and Simpson index. Inspection of funnel plots revealed some asymmetry (**Supplementary Figure S1**), but no statistically significant results were observed using Egger’s test (**Supplementary Table S10**).

**Figure 2:**
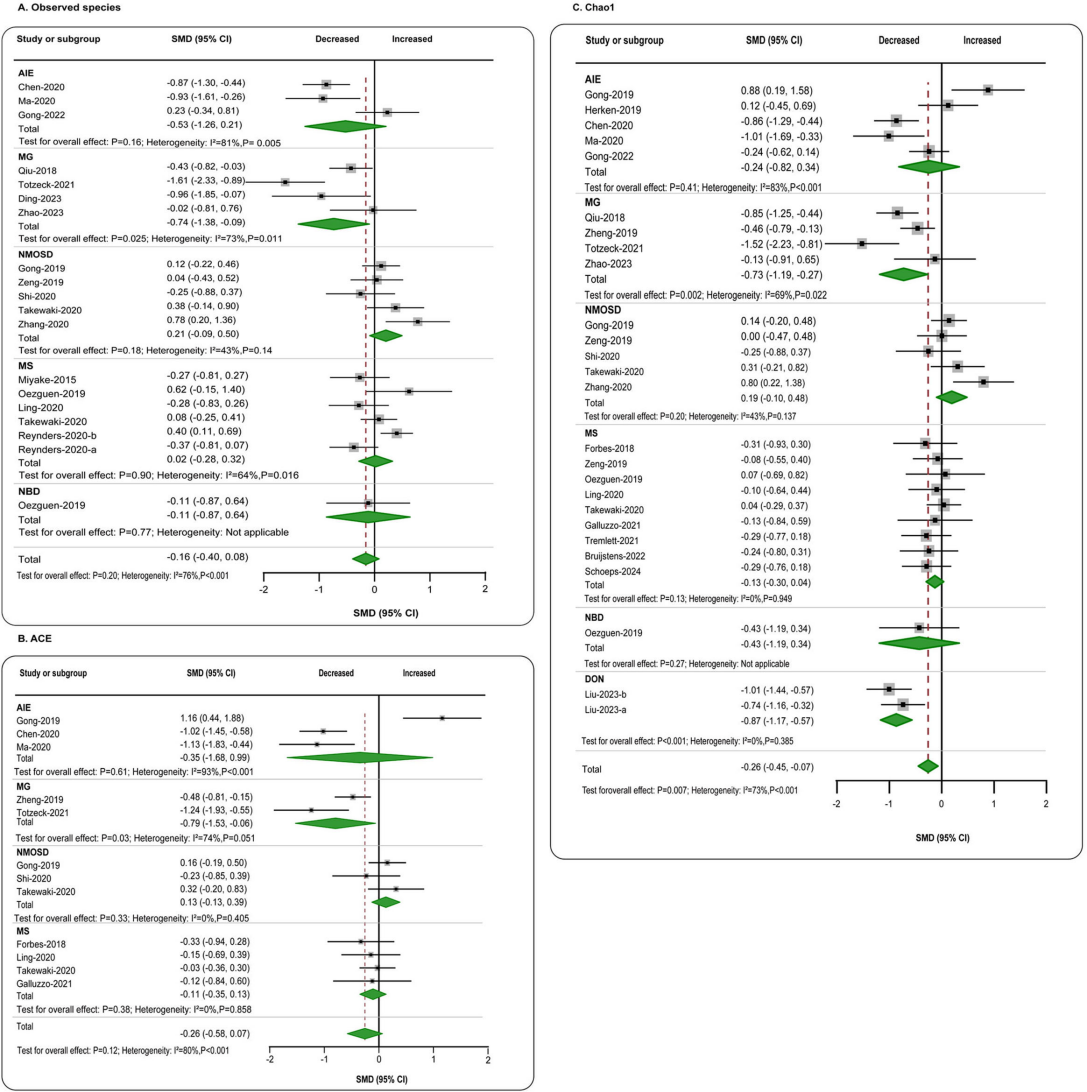
Forest plots of α-diversity richness estimators in the gut microbiota of patients with autoimmune neurological diseases compared with healthy controls. **(A)** Shannon index; **(B)** Simpson index. AIE, autoimmune encephalitis; MG, myasthenia gravis; NMOSD, optic neuromyelitis optica spectrum disorders; MS, multiple sclerosis; NBD, neuro-Behcet’s disease; DON, demyelinating optic neuritis; CI, confidence interval; NA, not applicable.

**Figure 3:**
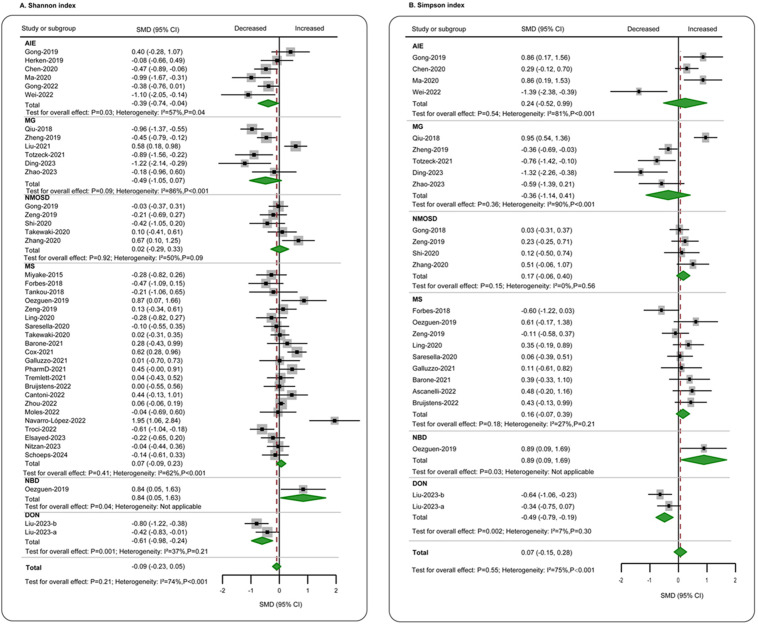
Forest plots of α-diversity evenness estimators in the gut microbiota of patients with autoimmune neurological diseases compared with healthy controls. **(A)** Shannon index; **(B)** Simpson index. AIE, autoimmune encephalitis; MG, myasthenia gravis; NMOSD, optic neuromyelitis optica spectrum disorders; MS, multiple sclerosis; NBD, neuro-Behcet’s disease; DON, demyelinating optic neuritis; CI, confidence interval; NA, not applicable.

In terms of richness, data on observed species in patients (*n* = 644) compared to healthy individuals (*n* = 773) were provided by 16 studies (11, 12, 21, 23, 25–27, 29–33, 35, 44, 49, 50). The aggregated assessment indicated no significant differences between the groups (SMD = −0.16, 95% CI = −0.40 to 0.08, *p* = 0.20), with considerable heterogeneity (*I* ^2^ = 76%). When analyzing individual diseases, a significant reduction in gut microbiota richness was found exclusively in MG patients (SMD = −0.74, 95% CI = −1.38 to −0.09, *p* = 0.025) (**Figure 2A**). ACE data were available from 11 studies (4, 5, 11, 21, 25, 29, 31, 32, 41, 49, 55), comparing patients with autoimmune neurological diseases (*n* = 390) to controls (*n* = 378). The overall estimate for combined autoimmune neurological diseases showed a decline in ACE compared to controls, albeit with a small effect size (SMD = −0.26, 95% CI = −0.58 to 0.07, *p* = 0.12; *I* ^2^ = 80%), which did not reach statistical significance. However, a moderate significant decrease was observed in MG patients individually (SMD = −0.79, 95% CI = −1.53 to −0.06, *p* = 0.03; *I* ^2^ = 74%) (**Figure 2B**). Chao1 data were reported in 22 studies (4–6, 11, 12, 21, 23, 25, 27, 29–33, 41, 44, 49, 55, 61, 63, 72, 73), encompassing patients with autoimmune neurological diseases (*n* = 942) and controls (*n* = 866). Overall, there was a significant reduction in Chao1, though the effect size was small (SMD = −0.26, 95% CI = −0.45 to −0.07, *p* < 0.01), with high heterogeneity (*I* ^2^ = 73%). When focusing on specific diseases, a significant reduction was also only found in MG (SMD = −0.73, 95% CI = −1.19 to −0.27, *p* < 0.01; *I* ^2^ = 69%) (**Figure 2C**). Regarding evenness, the Shannon index was reported in 38 studies (4–6, 11–13, 20, 21, 23–27, 29–33, 35, 41, 42, 44, 49, 51–53, 55, 60, 61, 63–69, 72, 73), comparing patients (*n* = 2,101) to controls (*n* = 1,754). The combined assessment revealed no significant difference (SMD = −0.09, 95% CI = −0.23 to 0.05, *p* = 0.21). When examining specific diseases, a significant decrease was only observed in the AIE group (SMD = −0.39, 95% CI = −0.74 to −0.04, *p* = 0.03; *I* ^2^ = 57%) (**Figure 3A**). Data on the Simpson index were supplied by 22 studies (4, 5, 11, 20, 21, 23, 25–27, 29–31, 33, 41, 44, 49, 51, 52, 55, 62, 63, 73), comparing patients (*n* = 712) to controls (*n* = 664). No significant change between the groups was observed (SMD = 0.07, 95% CI = −0.15 to 0.28, *p* = 0.48; *I* ^2^ = 75%) (**Figure 3B**).

To explore the reasons for the higher heterogeneity, subgroup analyses were conducted. In the subgroup analyses of Eastern and Western studies, a minor difference in the Shannon index was noted solely in the Eastern population (SMD = −0.27, 95% CI = −0.47 to −0.07, *p* < 0.01; *I* ^2^ = 72%) (**Supplementary Table S6**). Most α-diversity indices exhibited no significant difference, and substantial heterogeneity persisted (*I* ^2^ ranged from 46% to 85%). In the subgroup analyses of treatment-naive patients versus those on treatment, a decrease in most α-diversity indices was evident only in treatment-naive patients. Nevertheless, substantial heterogeneity remained (*I* ^2^ ranged from 44% to 82%) (**Supplementary Table S7**). Additionally, sensitivity analyses were conducted by excluding low-quality studies (**Supplementary Table S8**) and studies without variable matching (**Supplementary Table S9**). Consistent with the overall analysis, only Chao1 remained significantly decreased in individuals with autoimmune neurological diseases, indicating that the data are robust.

### 3.4 Synthesis of β-diversity

**Figure 4:**
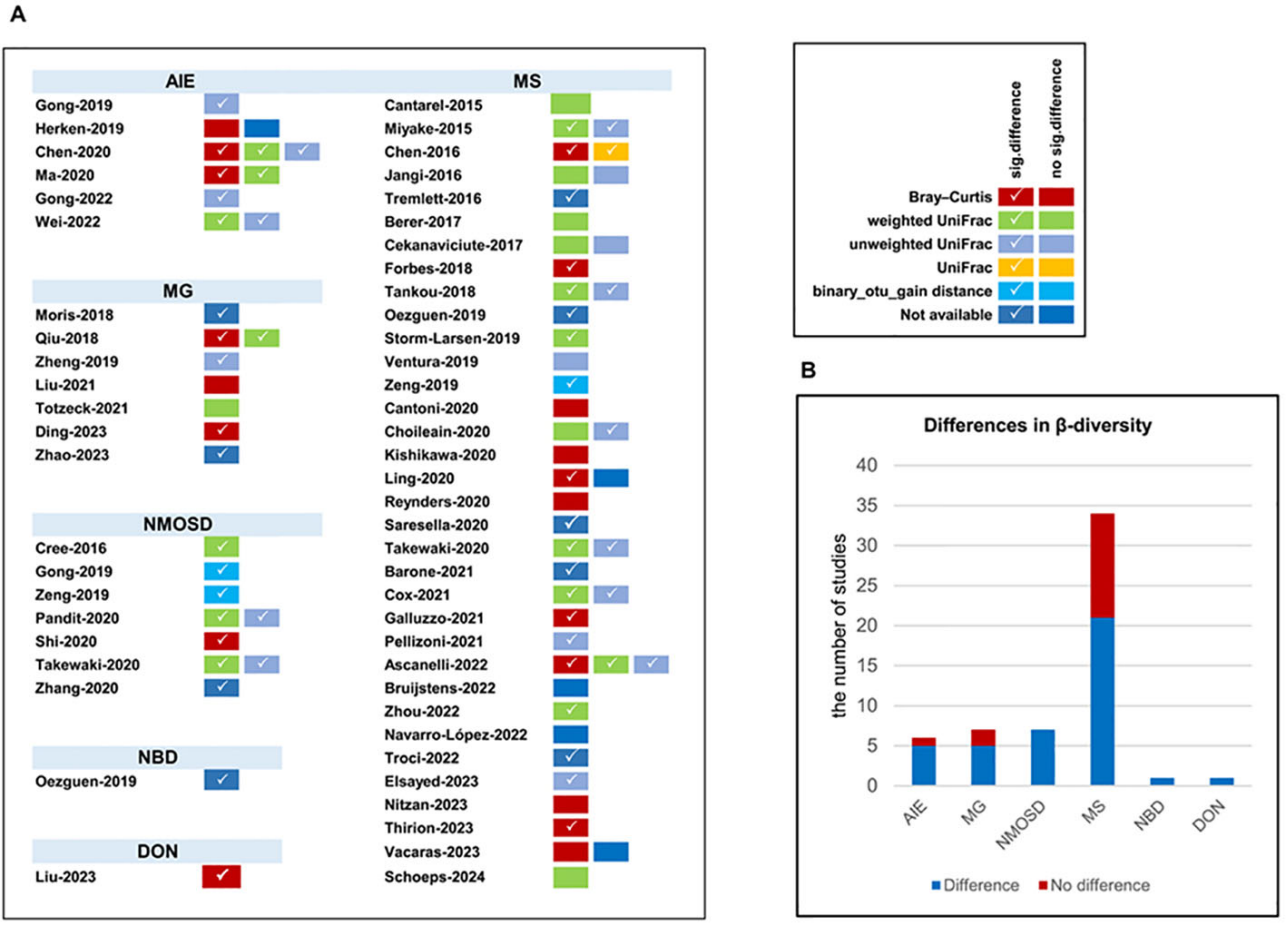
The β-diversity comparison between patients with autoimmune neurological diseases compared with healthy controls. **(A)** β-diversity comparison in included studies; **(B)** distribution of β-diversity in neurological autoimmune diseases. AIE, autoimmune encephalitis; MG, myasthenia gravis; NMOSD, optic neuromyelitis optica spectrum disorders; MS, multiple sclerosis; NBD, neuro-Behcet’s disease; DON, demyelinating optic neuritis. Number of studies: autoimmune encephalitis (AIE), 5/6; myasthenia gravis (MG), 5/7; optic neuromyelitis optica spectrum disorders (NMOSD), 7/7; multiple sclerosis (MS), 21/34; neuro-Behcet’s disease (NBD), 1/1; demyelinating optic neuritis (DON), 1/1.

A comparison of β-diversity between patients with autoimmune neurological disorders and healthy individuals was conducted across 53 studies encompassing six distinct diseases (AIE, MG, NMOSD, MS, NBD, and DON) using varied methodologies (**Figure 4**). Significant changes in β-diversity between patients with autoimmune neurological disorders and healthy individuals were reported in 4 out of 5 studies on anti-NMDAR encephalitis, 1 study on anti-LGI1 encephalitis, 5 out of 7 studies on MG, all 7 studies on NMOSD, 21 out of 34 studies on MS, 1 study on NBD, and 1 study on DON. Notably, among these disorders, only NMOSD showed consistently distinct β-diversity.

### 3.5 Synthesis of differentially abundant microbes

**Figure 5:**
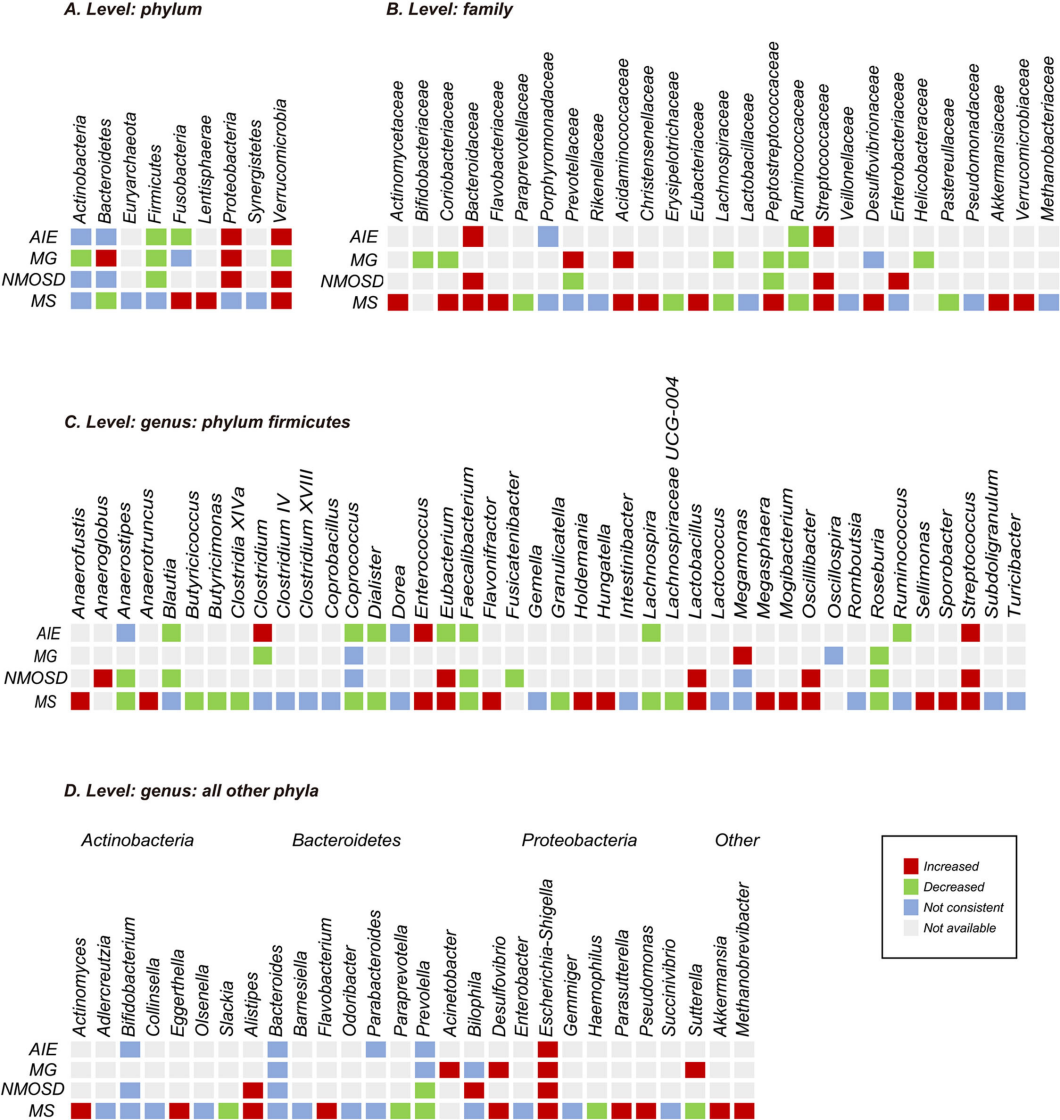
Changes in relative abundance of microbial taxa reported by at least two studies from a diagnostic category. **(A)** Phylum level; **(B)** family level; **(C)** genus level: phylum firmicutes; **(D)** genus level: all other phyla. All have been reported by more than one research group. The red and green grids indicate increase and decrease, respectively. Purple grids indicate inconsistency that was defined as less than 75% agreement between studies. Gray grids indicate not examined, not reported, or not replicated. Number of studies: autoimmune encephalitis (AIE), 6; myasthenia gravis (MG), 6; optic neuromyelitis optica spectrum disorders (NMOSD), 7; multiple sclerosis (MS), 35.

A total of 55 studies compared the relative abundance of gut microbiota at the genus, family, or phylum levels between patients with autoimmune neurological disorders and healthy individuals. These alterations encompassed 11 phyla, 32 families, and 88 genera after excluding non-replicated findings (**Supplementary Figure S2**). Within-disease and between-disease comparisons were summarized for diseases with sufficient research (AIE, MG, NNMOSD, and MS reported by two or more studies). The findings suggest that specific microorganisms undergo common changes across multiple autoimmune neurological diseases, although most consistent disease-related changes were replicated by only two studies. At the phylum level, the most consistent alterations were an increase in *Proteobacteria* (8 of 8 studies) and a decrease in *Firmicutes* (8 of 8 studies) in AIE, MG, and NMOSD, and an increase in *Verrucomicrobia* (9 of 10 studies) in AIE, NMOSD, and MS. At the family level, *Ruminococcaceae* (11 of 13 studies) and *Lachnospiraceae* (14 of 16 studies) consistently showed decreases, while *Streptococcaceae* showed an increase (10 of 11 studies) in AIE, MG, NMOSD, and MS. At the genus level, the most consistent alterations were an increase in *Streptococcus* (16 of 18 studies) and *Escherichia-Shigella* (12 of 13 studies), and a decrease in *Roseburia* (17 of 18 studies) and *Faecalibacterium* (23 of 24 studies) (**Figure 5**) in AIE, MG, NMOSD, and MS.

In subgroup analyses, microbial changes were found only in studies from Eastern countries, including an increase in genus *Acinetobacter* and decreases in genera *Clostridium_XIV*, *Granulicatella*, and *Megasphaera*. In contrast, decreases in the genus *Slackia* and increases in the genera *Holdemania*, *Megasphaera*, and *Olsenella* were observed in studies from Western countries. In subgroup analyses of immunotherapy use, comparisons were made between studies without treatment (*n* = 22) and those with 80% or more of patients on medication (*n* = 13). Increases in the genera *Acinetobacter*, *Collinsella*, *Enterococcus*, *Escherichia*, *Fusicatenibacter*, *Succinivibrio*, and *Sutterella*, along with decreases in the family *Rikenellaceae* and the genera *Gemella* and *Haemophilus*, were exclusively reported in treatment-naive studies. However, these findings should be regarded as preliminary due to the uneven distribution of studies by region (e.g., AIE, MG, and NMOSD were predominantly studied in Eastern populations) and immunotherapy use (e.g., AIE and MG were predominantly studied in treatment-naive patients, while NMOSD was predominantly studied in patients receiving immunotherapy).

## 4 Discussion

Our review undertook a comprehensive assessment of the alterations observed in the intestinal microbiota associated with autoimmune neurological diseases. We included 62 studies covering six neurological autoimmune illnesses, indicating a correlation between gut microbiota dysbiosis and autoimmune neurological disorders in general. The findings predominantly reflect non-specific, shared microbial changes rather than disease-specific patterns. We found that (1) there were no significant alterations in overall α-diversity indices across autoimmune neurological diseases, though a reduction in α-diversity was noted in treatment-naive patients; (2) although most studies reported significant variations in β-diversity, only NMOSD consistently exhibited differences; and (3) a reduction in SCFA-producing bacteria, including *Faecalibacterium* and *Roseburia*, was observed in patients with AIE, MG, NMOSD, and MS, alongside an increase in pathogenic or opportunistic pathogens, including *Streptococcus* and *Escherichia-Shigella*. Disease-specific microbial changes were not detected, and even when specific microbial changes were observed, they were rarely replicated across studies.

A decrease in α-diversity is commonly observed in various diseases and is believed to contribute to their development. Higher α-diversity is generally seen as an indicator of better gut health. However, no significant reduction in α-diversity was detected in autoimmune neurological diseases as a whole, with the exception of MG. A meta-analysis by PLASSAIS et al. also noted that gut microbiota α-diversity does not serve as a biomarker for MS and Parkinson’s disease (14). It appears that α-diversity is an unreliable biomarker for autoimmune neurological diseases. However, as noted by Ashley Shade, the oversimplified view that “higher diversity is better” overlooks the intricate mechanisms underlying microbiota and host interactions (74). The α-diversity is affected by numerous factors, including measurement methods, environment, and medication use (75). In subgroup analyses of studies focusing on untreated patients, slight decreases in α-diversity were observed, suggesting that patients with autoimmune neurological diseases experience gut dysbiosis, and immunotherapy may recover the microbial diversity. However, these findings should be viewed with caution given the considerable heterogeneity and potential publication bias among the included studies. The β-diversity analyses consistently demonstrated distinct patterns in each autoimmune neurological disease compared to healthy controls. However, it remains unclear whether autoimmune neurological diseases differ significantly among themselves or from other diseases. Only five studies conducted cross-diagnostic comparisons of β-diversity, revealing no significant differences (25, 28, 30, 32, 41, 44). Furthermore, these analyses did not specify the nature or extent of the differences. Further research is required to effectively utilize diversity as a diagnostic tool.

The brain–gut axis facilitates bidirectional gut–brain regulation through a variety of processes such as chemical signaling, neuronal pathways, and immunological modulation (76). Among these, immunomodulatory effects play a crucial role. Gut microbiota not only regulates the peripheral immune response by disrupting gut barrier integrity and altering metabolite synthesis but also participates in the immune response of the central nervous system. It influences the development, maturation, and activation of microglia and increases the permeability of the blood–brain barrier (BBB). Disruption of the intestinal barrier can cause systemic inflammation by disrupting T- and B-cell homeostasis. Additionally, microbial metabolites, such as short-chain fatty acids (SCFAs), exhibit direct immunomodulatory effects (77). Our findings indicate that similar microbial changes may be shared across autoimmune neurological diseases. *Roseburia* and *Faecalibacterium* were reduced, whereas *Streptococcus* and *Escherichia-Shigella* were elevated in patients with AIE, MG, NMOSD, and MS. *Faecalibacterium* and *Roseburia* are known producers of SCFAs, notably butyrate. *Faecalibacterium*, a Gram-positive bacterium with potent anti-inflammatory properties, has been shown to suppress TNF-α expression and alleviate inflammation in various immunological and neurological diseases (78, 79). *Roseburia* generates SCFAs, mainly butyrate, by the fermentation of dietary fibers, and has been observed to decrease in various disorders (80). SCFAs play multifaceted roles in affecting gastrointestinal physiology, peripheral immunity, activation of microglia, and BBB integrity, thereby indirectly influencing brain function. Research has shown that SCFAs can initiate metabolic and epigenetic reprogramming, potentially attenuating inflammatory immune responses (81). In the included studies, patients with autoimmune neurological diseases exhibited significantly lower levels of SCFAs in both their blood and feces compared to healthy individuals (13, 22–24, 29–31, 51, 64, 65). SCFAs can alleviate clinical symptoms by increasing IL-10 production and inhibiting T helper 17 (Th17) cells (82). In contrast, *Streptococcus*, known for its association with inflammation, is positively correlated with Th17 cell and negatively with regulatory T cells. It also produces neurotoxins, such as streptokinase and streptomycin, which may cause irreversible neuronal damage (83). *Streptococcus* level was reported to be increased in immunotherapy-naive studies, but decreased in treatment studies (59, 62, 78). Similarly, *Escherichia-Shigella* has been associated with proinflammatory processes in various disorders, including acute intestinal damage, increased BBB permeability, and neuroinflammation. The findings indicate a decrease in SCFA-producing bacteria, including *Roseburia* and *Faecalibacterium*, alongside an increase in pathogenic or opportunistic pathogens including *Streptococcus* and *Escherichia-Shigella* in patients with autoimmune neurological diseases. These consistent inflammation-associated changes in gut flora across autoimmune neurological diseases may elucidate shared pathophysiological mechanisms and potentially exacerbate persistent inflammation within the nervous system.

Interestingly, *Akkermansia* has shown potential in alleviating various diseases such as obesity, diabetes, and epilepsy, and has been considered a potential next-generation probiotic (84–86). However, its role in autoimmune neurological disorders remains ambiguous. Elevated *Akkermansia* level has been reported in patients with MS and NMOSD, with several studies indicating a positive correlation between its abundance with disease severity (51, 62, 87). Extracts of *Akkermansia muciniphila* have been shown to promote T-cell differentiation into Th1 cells, contributing to proinflammatory responses (9). Additionally, Vallino et al. reported that the cerebrospinal fluid of patients with MS contained higher levels of anti-*A. muciniphila* IgG than those of healthy individuals (88). Conversely, Cox et al. reported a negative correlation between *Akkermansia* abundance and disability, as well as T2 lesion volume, while observing a positive correlation with brain volume in MS patients. Furthermore, MS-derived *Akkermansia* was found to improve experimental autoimmune encephalomyelitis via lowering RORγt+ and IL-17-producing γδ T cells, indicating a potentially beneficial, compensatory response of the microbiome in MS (53). Liu and colleagues further demonstrated that administering miR-30d orally in mice augmented Treg counts and alleviated symptoms of experimental autoimmune encephalomyelitis by enhancing *Akkermansia* level in the gastrointestinal tract (89). *Akkermansia*’s anti-inflammatory properties may be strain-specific, as suggested by recent findings (90). With advancements in technology, its role in autoimmune neurological diseases may become better understood.

Various clinical and demographic factors may account for the inconsistencies observed across studies. Based on current evidence, we concentrated on two key aspects: geographical region and immunotherapy. Geographical region, along with diet as a related factor, play a significant role in shaping the composition of the microbiota (91). Our analysis revealed that some microbiota changes appear to be population-specific, such as higher levels of *Megasphaera* and *Olsenella* in Western populations and *Acinetobacter* in Eastern populations. We also recognize significant regional variations in disease incidence rates. MS, which has a higher global incidence, is more prevalent in Western countries, leading to greater representation in the studies analyzed. Conversely, NMOSD, AIE, and MG are less common, with NMOSD showing a higher incidence among non-White populations, driving focused research efforts in regions like China (92–94). These disparities may influence the analysis and limit the generalizability of the findings. Furthermore, we observed microbiota alterations associated with immunotherapy, such as decreased *Streptococcus* and increased *Prevotella*. To better differentiate between the effects of confounders and genuine disease-related changes, it is imperative that forthcoming studies provide exhaustive data encompassing all predominant microbial taxa, as well as variations across diverse countries and regions.

Our findings suggest that disturbances in the gut microbiota are related to autoimmune neurological diseases, with shared microbiota changes observed across different conditions. However, some limitations should be acknowledged. Firstly, our review may be subject to language bias, as only English-language studies were included, potentially excluding significant research from non-English-speaking regions. Secondly, this review focused solely on gut microbiota composition without establishing causality. Geographical differences were observed in the studies, with MS predominantly studied in Western countries and AIE, MG, and NMOSD predominantly studied in China. While the categorization of countries into Eastern and Western regions serves as a basic method to address differences in diet and genetics, this approach remains crude. Additionally, most studies had relatively small to medium sample sizes, suggesting that analysis may still be preliminary and inconclusive. At the same time, differences in the methods of analysis can also lead to variations in results. Furthermore, given that the majority of research encompassed individuals receiving diverse immunotherapies, as well as those not on any treatment, inconsistency in data collection on covariates among studies and the lack of control for possible confounders in some analyses also pose limitations (95). As research is conducted on larger samples, encompassing a wider range of regions and population groups, and with increasing availability of metadata, there will be a corresponding enhancement in the reliability of findings. Concurrently, it is recommended that further studies should include longitudinal studies to elucidate the chronological interactions among gut microbiota, disease evolution, and therapeutic strategies. Finally, it is noteworthy that most included studies utilized 16s rRNA sequencing to analyze intestinal flora. As the field evolves, employing alternative histologic techniques will facilitate a deeper exploration of the gut microbiota beyond compositional analysis. Notwithstanding the aforementioned limitations, the findings from the majority of studies provide a critical foundation for understanding the role of gut microbiota in autoimmune neurological disorders.

## 5 Conclusions

This study provides the first comprehensive assessment of gut microbiota changes in autoimmune neurological disorders. The existing evidence suggests that gut microbiota dysbiosis is associated with these conditions, primarily due to potential non-specific changes in microorganisms, including decreased SCFA-producing bacteria with anti-inflammatory capacity, and increased pathogenic or opportunistic pathogens. These insights provide a promising foundation for diagnosing these diseases and developing novel therapeutic strategies targeting the gut microbiota to manage or even prevent these conditions.

## Data Availability

The original contributions presented in the study are included in the article/**Supplementary Material**. Further inquiries can be directed to the corresponding author.
