# Supplementary material for: Perturbations in gut microbiota composition in patients with autoimmune neurological diseases: a systematic review and meta-analysis
Source: Front Immunol. 2025 Feb 6;16:1513599. doi: 10.3389/fimmu.2025.1513599 (PMC11839609; doi:10.3389/fimmu.2025.1513599)
Supplement: Supplementary file 1 [file DataSheet1.pdf]

Supplementary Material

Supplementary Table 1. Systematic search details

Supplementary Table 2. Summary of Exclusion Criteria for Full-Text Articles

Supplementary Table 3. Detailed characteristics of the included studies

Supplementary Table 4. Quality assessment of the included studies using the Newcastle-Ottawa Scale (NOS)

Supplementary Table 5. Methodology of stool processing of the included studies

Supplementary Table 6. Results of subgroup analyses of different study regions

Supplementary Table 7. Results of subgroup analyses of studies with patients on treatment or treatment-naive studies

Supplementary Table 8. Results of sensitivity analyses by excluding studies with low quality (Newcastle-Ottawa Scale ≤5)

Supplementary Table 9. Results of sensitivity analyses by excluding studies without matching any variables

Supplementary Table 10. Publication bias assessment by egger's regression test in alpha diversity

Supplementary Figure 1. Funnel plots of publication bias assessment in alpha diversity

Supplementary Figure 2. Figures for study-level findings of relative abundance of gut microbes

Supplementary Table 1. Systematic search details ----- performed on March 1, 2024

1.1. PubMed

Step	Search	Hits
#1	((Anti-N-Methyl-D-Aspartate Receptor Encephalitis[MeSH Terms]) OR (Anti N Methyl D Aspartate Receptor Encephalitis[Title/Abstract] OR Anti-N-Methyl-D-Aspartate Receptor Encephalitides[Title/Abstract] OR Encephalitides, Anti-N-Methyl-D-Aspartate Receptor[Title/Abstract] OR Encephalitis, Anti-N-Methyl-D-Aspartate Receptor[Title/Abstract] OR Anti-NMDA Receptor Encephalitis[Title/Abstract] OR Anti NMDA Receptor Encephalitis[Title/Abstract] OR Anti-NMDA Receptor Encephalitides[Title/Abstract] OR Encephalitides, Anti-NMDA Receptor[Title/Abstract] OR Encephalitis, Anti-NMDA Receptor[Title/Abstract] OR Receptor Encephalitides, Anti-NMDA[Title/Abstract] OR Receptor Encephalitis, Anti-NMDA[Title/Abstract] OR Anti-NMDAR Encephalitis[Title/Abstract] OR Anti NMDAR Encephalitis[Title/Abstract] OR Anti-NMDAR Encephalitides[Title/Abstract] OR Encephalitides, Anti-NMDAR[Title/Abstract] OR Encephalitis, Anti-NMDAR[Title/Abstract] OR Non-paraneoplastic Anti-N-Methyl-D-Aspartate Receptor Encephalitis[Title/Abstract] OR Non paraneoplastic Anti N Methyl D Aspartate Receptor Encephalitis[Title/Abstract] OR Non-paraneoplastic Anti-NMDA Receptor Encephalitis[Title/Abstract] OR Non paraneoplastic Anti NMDA Receptor Encephalitis[Title/Abstract] OR Non-paraneoplastic Anti-NMDAR Encephalitis[Title/Abstract] OR Anti-NMDAR Encephalitides, Non-paraneoplastic[Title/Abstract] OR Anti-NMDAR Encephalitis, Non-paraneoplastic[Title/Abstract] OR Encephalitides, Non-paraneoplastic Anti-NMDAR[Title/Abstract] OR Encephalitis, Non-paraneoplastic Anti-NMDAR[Title/Abstract] OR Non paraneoplastic Anti NMDAR Encephalitis[Title/Abstract] OR Non-paraneoplastic Anti-NMDAR Encephalitides[Title/Abstract] OR Paraneoplastic Anti-N-Methyl-D-Aspartate Receptor Encephalitis[Title/Abstract] OR Paraneoplastic Anti N Methyl D Aspartate Receptor Encephalitis[Title/Abstract] OR Paraneoplastic Anti-NMDA Receptor Encephalitis[Title/Abstract] OR Paraneoplastic Anti NMDA Receptor Encephalitis[Title/Abstract] OR Paraneoplastic Anti-NMDAR Encephalitis[Title/Abstract] OR Anti-NMDAR Encephalitides, Paraneoplastic[Title/Abstract] OR Anti-NMDAR Encephalitis, Paraneoplastic[Title/Abstract] OR Encephalitides, Paraneoplastic Anti-NMDAR[Title/Abstract] OR Encephalitis, Paraneoplastic Anti-NMDAR[Title/Abstract] OR Paraneoplastic Anti NMDAR Encephalitis[Title/Abstract] OR Paraneoplastic Anti-NMDAR Encephalitides[Title/Abstract])) AND ((Gastrointestinal Microbiome[MeSH Terms]) OR (Gut Microbiome[Title/Abstract] OR Gut Microbiomes[Title/Abstract] OR Gut Microflora[Title/Abstract] OR Gut Microbiota[Title/Abstract]	8

	OR Gut Microbiotas[Title/Abstract] OR Gastrointestinal Flora[Title/Abstract] OR Gut Flora[Title/Abstract] OR Gastrointestinal Microbiota[Title/Abstract] OR Gastrointestinal Microbiotas[Title/Abstract] OR Gastrointestinal Microbial Community[Title/Abstract] OR Gastrointestinal Microbial Communities[Title/Abstract] OR Gastrointestinal Microflora[Title/Abstract] OR Gastric Microbiome[Title/Abstract] OR Gastric Microbiomes[Title/Abstract] OR Intestinal Microbiome[Title/Abstract] OR Intestinal Microbiomes[Title/Abstract] OR Intestinal Microbiota[Title/Abstract] OR Intestinal Microbiotas[Title/Abstract] OR Intestinal Microflora[Title/Abstract] OR Intestinal Flora[Title/Abstract] OR Enteric Bacteria[Title/Abstract]))	
#2	(anti-leucine-rich glioma-inactivated 1[MeSH Terms]) OR (LGI1[Title/Abstract] OR Anti-LGI1[Title/Abstract])AND ((Gastrointestinal Microbiome[MeSH Terms]) OR (Gut Microbiome[Title/Abstract] OR Gut Microbiomes[Title/Abstract] OR Gut Microflora[Title/Abstract] OR Gut Microbiota[Title/Abstract] OR Gut Microbiotas[Title/Abstract] OR Gastrointestinal Flora[Title/Abstract] OR Gut Flora[Title/Abstract] OR Gastrointestinal Microbiota[Title/Abstract] OR Gastrointestinal Microbiotas[Title/Abstract] OR Gastrointestinal Microbial Community[Title/Abstract] OR Gastrointestinal Microbial Communities[Title/Abstract] OR Gastrointestinal Microflora[Title/Abstract] OR Gastric Microbiome[Title/Abstract] OR Gastric Microbiomes[Title/Abstract] OR Intestinal Microbiome[Title/Abstract] OR Intestinal Microbiomes[Title/Abstract] OR Intestinal Microbiota[Title/Abstract] OR Intestinal Microbiotas[Title/Abstract] OR Intestinal Microflora[Title/Abstract] OR Intestinal Flora[Title/Abstract] OR Enteric Bacteria[Title/Abstract]))	1
#3	((Myasthenia Gravis[MeSH Terms]) OR (Myasthenia Gravis[Title/Abstract] OR Myasthenia Gravis, Ocular[Title/Abstract] OR Ocular Myasthenia Gravis[Title/Abstract] OR Myasthenia Gravis, Generalized[Title/Abstract] OR Generalized Myasthenia Gravis[Title/Abstract] OR Muscle-Specific Receptor Tyrosine Kinase Myasthenia Gravis[Title/Abstract] OR Muscle Specific Receptor Tyrosine Kinase Myasthenia Gravis[Title/Abstract] OR Muscle-Specific Tyrosine Kinase Antibody Positive Myasthenia Gravis[Title/Abstract] OR Muscle Specific Tyrosine Kinase Antibody Positive Myasthenia Gravis[Title/Abstract] OR MuSK MG[Title/Abstract] OR MuSK Myasthenia Gravis[Title/Abstract] OR Myasthenia Gravis, MuSK[Title/Abstract] OR Anti-MuSK Myasthenia Gravis[Title/Abstract] OR Anti MuSK Myasthenia Gravis[Title/Abstract] OR Myasthenia Gravis, Anti-MuSK[Title/Abstract])) AND ((Gastrointestinal Microbiome[MeSH Terms]) OR (Gut Microbiome[Title/Abstract] OR Gut Microbiomes[Title/Abstract] OR Gut Microflora[Title/Abstract] OR Gut Microbiota[Title/Abstract] OR Gut Microbiotas[Title/Abstract] OR Gastrointestinal Flora[Title/Abstract] OR Gut Flora[Title/Abstract] OR Gastrointestinal Microbiota[Title/Abstract] OR Gastrointestinal Microbiotas[Title/Abstract] OR Gastrointestinal Microbial Community[Title/Abstract] OR Gastrointestinal Microbial Communities[Title/Abstract] OR Gastrointestinal Microflora[Title/Abstract] OR Gastric Microbiome[Title/Abstract] OR Gastric Microbiomes[Title/Abstract] OR Intestinal Microbiome[Title/Abstract] OR Intestinal Microbiomes[Title/Abstract] OR Intestinal Microbiota[Title/Abstract] OR Intestinal	36

	Microbiotas[Title/Abstract] OR Intestinal Microflora[Title/Abstract] OR Intestinal Flora[Title/Abstract] OR Enteric Bacteria[Title/Abstract]))	
#4	((Neuromyelitis Optica[MeSH Terms]) OR (NMO Spectrum Disorder[Title/Abstract] OR NMO Spectrum Disorders[Title/Abstract] OR Neuromyelitis Optica (NMO) Spectrum Disorder[Title/Abstract] OR Neuromyelitis Optica Spectrum Disorders[Title/Abstract] OR Devic Neuromyelitis Optica[Title/Abstract] OR Devic Neuromyelitis Opticas[Title/Abstract] OR Neuromyelitis Optica, Devic[Title/Abstract] OR Neuromyelitis Opticas, Devic[Title/Abstract] OR Devic's Disease[Title/Abstract] OR Devics Disease[Title/Abstract] OR Disease, Devic's[Title/Abstract] OR Devic Disease[Title/Abstract] OR Disease, Devic[Title/Abstract] OR Devic Syndrome[Title/Abstract] OR Syndrome, Devic[Title/Abstract] OR Devic's Syndrome[Title/Abstract] OR Devics Syndrome[Title/Abstract] OR Syndrome, Devic's[Title/Abstract] OR Devic's Neuromyelitis Optica[Title/Abstract] OR Devics Neuromyelitis Optica[Title/Abstract] OR Neuromyelitis Optica, Devic's[Title/Abstract] OR Neuromyelitis Optica Spectrum Disorder[Title/Abstract] OR Neuromyelitis Optica (NMO) Spectrum Disorders[Title/Abstract])) AND ((Gastrointestinal Microbiome[MeSH Terms]) OR (Gut Microbiome[Title/Abstract] OR Gut Microbiomes[Title/Abstract] OR Gut Microflora[Title/Abstract] OR Gut Microbiota[Title/Abstract] OR Gut Microbiotas[Title/Abstract] OR Gastrointestinal Flora[Title/Abstract] OR Gut Flora[Title/Abstract] OR Gastrointestinal Microbiota[Title/Abstract] OR Gastrointestinal Microbiotas[Title/Abstract] OR Gastrointestinal Microbial Community[Title/Abstract] OR Gastrointestinal Microbial Communities[Title/Abstract] OR Gastrointestinal Microflora[Title/Abstract] OR Gastric Microbiome[Title/Abstract] OR Gastric Microbiomes[Title/Abstract] OR Intestinal Microbiome[Title/Abstract] OR Intestinal Microbiomes[Title/Abstract] OR Intestinal Microbiota[Title/Abstract] OR Intestinal Microbiotas[Title/Abstract] OR Intestinal Microflora[Title/Abstract] OR Intestinal Flora[Title/Abstract] OR Enteric Bacteria[Title/Abstract]))	17
#5	((Multiple Sclerosis[MeSH Terms]) OR (Sclerosis, Multiple[Title/Abstract] OR Sclerosis, Disseminated[Title/Abstract] OR Disseminated Sclerosis[Title/Abstract] OR MS (Multiple Sclerosis[Title/Abstract]) OR Multiple Sclerosis, Acute Fulminating[Title/Abstract])) AND ((Gastrointestinal Microbiome[MeSH Terms]) OR (Gut Microbiome[Title/Abstract] OR Gut Microbiomes[Title/Abstract] OR Gut Microflora[Title/Abstract] OR Gut Microbiota[Title/Abstract] OR Gut Microbiotas[Title/Abstract] OR Gastrointestinal Flora[Title/Abstract] OR Gut Flora[Title/Abstract] OR Gastrointestinal Microbiota[Title/Abstract] OR Gastrointestinal Microbiotas[Title/Abstract] OR Gastrointestinal Microbial Community[Title/Abstract] OR Gastrointestinal Microbial Communities[Title/Abstract] OR Gastrointestinal Microflora[Title/Abstract] OR Gastric Microbiome[Title/Abstract] OR Gastric Microbiomes[Title/Abstract] OR Intestinal Microbiome[Title/Abstract] OR Intestinal Microbiomes[Title/Abstract] OR Intestinal Microbiota[Title/Abstract] OR Intestinal Microbiotas[Title/Abstract] OR Intestinal Microflora[Title/Abstract] OR Intestinal Flora[Title/Abstract] OR Enteric Bacteria[Title/Abstract]))	466
#6	((Encephalomyelitis, Acute Disseminated[MeSH Terms]) OR (Acute Disseminated Encephalomyelitis[Title/Abstract] OR Acute Disseminated	211

	Encephalomyelitides[Title/Abstract] OR Disseminated Encephalomyelitides, Acute[Title/Abstract] OR Encephalomyelitides, Acute Disseminated[Title/Abstract] OR Disseminated Encephalomyelitis, Acute[Title/Abstract] OR Encephalomyelitis, Postexanthem[Title/Abstract] OR Postexanthem Encephalomyelitis[Title/Abstract] OR Postinfectious Encephalomyelitis[Title/Abstract] OR Encephalomyelitis, Postinfectious[Title/Abstract] OR Encephalitis, Post-Vaccinal[Title/Abstract] OR Encephalitis, Post Vaccinal[Title/Abstract] OR Encephalitis, Postvaccinal[Title/Abstract] OR Postvaccinal Encephalitis[Title/Abstract] OR Post-Vaccinal Encephalomyelitis[Title/Abstract] OR Encephalomyelitides, Post-Vaccinal[Title/Abstract] OR Encephalomyelitis, Post-Vaccinal[Title/Abstract] OR Post Vaccinal Encephalomyelitis[Title/Abstract] OR Post-Vaccinal Encephalomyelitides[Title/Abstract] OR Encephalitis, Vaccination[Title/Abstract] OR Vaccination Encephalitis[Title/Abstract] OR Post-Vaccinal Encephalitis[Title/Abstract] OR Encephalitides, Post-Vaccinal[Title/Abstract] OR Post Vaccinal Encephalitis[Title/Abstract] OR Post-Vaccinal Encephalitides[Title/Abstract])) AND ((Gastrointestinal Microbiome[MeSH Terms]) OR (Gut Microbiome[Title/Abstract] OR Gut Microbiomes[Title/Abstract] OR Gut Microflora[Title/Abstract] OR Gut Microbiota[Title/Abstract] OR Gut Microbiotas[Title/Abstract] OR Gastrointestinal Flora[Title/Abstract] OR Gut Flora[Title/Abstract] OR Gastrointestinal Microbiota[Title/Abstract] OR Gastrointestinal Microbiotas[Title/Abstract] OR Gastrointestinal Microbial Community[Title/Abstract] OR Gastrointestinal Microbial Communities[Title/Abstract] OR Gastrointestinal Microflora[Title/Abstract] OR Gastric Microbiome[Title/Abstract] OR Gastric Microbiomes[Title/Abstract] OR Intestinal Microbiome[Title/Abstract] OR Intestinal Microbiomes[Title/Abstract] OR Intestinal Microbiota[Title/Abstract] OR Intestinal Microbiotas[Title/Abstract] OR Intestinal Microflora[Title/Abstract] OR Intestinal Flora[Title/Abstract] OR Enteric Bacteria[Title/Abstract]))	

1.2. EMBASE

**Step**	Search	Hits
#1	('gastrointestinal microbiome':ab,ti OR 'gut microbiome':ab,ti OR 'gut microbiomes':ab,ti OR 'gut microflora':ab,ti OR 'gut microbiota':ab,ti OR 'gut microbiotas':ab,ti OR 'gastrointestina flora':ab,ti OR 'gut flora':ab,ti OR 'gastrointestinal microbiota':ab,ti OR 'gastrointestinal microbiotas':ab,ti OR 'gastrointestinal microbial community':ab,ti OR 'gastrointestinal microbial communities':ab,ti OR 'gastrointestinal microflora':ab,ti OR 'gastric microbiome':ab,ti OR 'gastric microbiomes':ab,ti OR 'intestinal microbiome':ab,ti OR 'intestinal microbiomes':ab,ti OR 'intestinal microbiota':ab,ti OR 'intestinal microbiotas':ab,ti OR 'intestinal microflora':ab,ti OR 'intestinal flora':ab,ti OR 'enteric bacteria':ab,ti)	95769
#2	('anti-n-methyl-d-aspartate receptor encephalitis':ab,ti OR 'anti n methyl d aspartate receptor encephalitis':ab,ti OR 'anti-n-methyl-d-aspartate	2,849

	receptor encephalitides':ab,ti OR 'encephalitides, anti-n-methyl-d-aspartate receptor':ab,ti OR 'encephalitis, anti-n-methyl-d-aspartate receptor':ab,ti OR 'anti-nmda receptor encephalitis':ab,ti OR 'anti nmda receptor encephalitis':ab,ti OR 'anti-nmda receptor encephalitides':ab,ti OR 'encephalitides, anti-nmda receptor':ab,ti OR 'encephalitis, anti-nmda receptor':ab,ti OR 'receptor encephalitides, anti-nmda':ab,ti OR 'receptor encephalitis, anti-nmda':ab,ti OR 'anti-nmda encephalitis':ab,ti OR 'anti nmdar encephalitis':ab,ti OR 'anti-nmdar encephalitides':ab,ti OR 'encephalitides, anti-nmdar':ab,ti OR 'encephalitis, anti-nmdar':ab,ti OR 'non-paraneoplastic anti-n-methyl-d-aspartate receptor encephalitis':ab,ti OR 'non paraneoplastic anti n methyl d aspartate receptor encephalitis':ab,ti OR 'non-paraneoplastic anti-nmda receptor encephalitis':ab,ti OR 'non paraneoplastic anti nmda receptor encephalitis':ab,ti OR 'non-paraneoplastic anti-nmdar encephalitis':ab,ti OR 'anti-nmdar encephalitides, non-paraneoplastic':ab,ti OR 'anti-nmdar encephalitis, non-paraneoplastic':ab,ti OR 'encephalitides, non-paraneoplastic anti-nmdar':ab,ti OR 'encephalitis, non-paraneoplastic anti-nmdar':ab,ti OR 'non paraneoplastic anti nmdar encephalitis':ab,ti OR 'non-paraneoplastic anti-nmdar encephalitides':ab,ti OR 'paraneoplastic anti-n-methyl-d-aspartate receptor encephalitis':ab,ti OR 'paraneoplastic anti n methyl d aspartate receptor encephalitis':ab,ti OR 'paraneoplastic anti-nmda receptor encephalitis':ab,ti OR 'paraneoplastic anti nmda receptor encephalitis':ab,ti OR 'paraneoplastic anti-nmdar encephalitis':ab,ti OR 'anti-nmdar encephalitides, paraneoplastic':ab,ti OR 'anti-nmdar encephalitis, paraneoplastic':ab,ti OR 'encephalitides, paraneoplastic anti-nmdar':ab,ti OR 'encephalitis, paraneoplastic anti-nmdar':ab,ti OR 'paraneoplastic anti nmdar encephalitis':ab,ti OR 'paraneoplastic anti-nmdar encephalitides':ab,ti)	
#3	#1 AND #2	5
#4	('anti caspr2':ab,ti OR caspr2:ab,ti OR 'anti-contactin associated protein-like 2':ab,ti OR 'anti-cntnap2 caspr2':ab,ti OR 'anti cntnap2':ab,ti)	1004
#5	#1 AND #4	0
#6	('anti-leucine-rich glioma-inactivated 1':ab,ti OR lgi1:ab,ti OR 'anti lgi1':ab,ti)	1660
#7	#1 AND #6	1
#8	('myasthenia gravis':ab,ti OR 'myasthenia gravis ocular':ab,ti OR 'ocular myasthenia gravis':ab,ti OR 'myasthenia gravis, generalized':ab,ti OR 'generalized myasthenia gravis':ab,ti OR 'muscle-specific receptor tyrosine kinase myasthenia gravis':ab,ti OR 'muscle specific receptor tyrosine kinase myasthenia gravis':ab,ti OR 'muscle-specific tyrosine kinase antibody positive myasthenia gravis':ab,ti OR 'muscle specific tyrosine kinase antibody positive myasthenia gravis':ab,ti OR 'musk mg':ab,ti OR 'musk myasthenia gravis':ab,ti OR 'myasthenia gravis, musk':ab,ti OR 'anti-musk myasthenia gravis':ab,ti OR 'anti musk myasthenia gravis':ab,ti OR 'myasthenia gravis, anti-musk':ab,ti)	18887
#9	#1 AND #8	34
#10	(('nmo spectrum disorder':ab,ti OR 'nmo spectrum disorders':ab,ti OR 'neuromyelitis optica':ab,ti) AND nmo:ab,ti AND 'spectrum disorder':ab,ti OR	809

	'neuromyelitis optica spectrum disorders':ab,ti OR 'devic neuromyelitis optica':ab,ti OR 'devic neuromyelitis opticas':ab,ti OR 'neuromyelitis optica devic':ab,ti OR 'neuromyelitis opticas devic':ab,ti OR 'devics disease':ab,ti OR 'disease devics':ab,ti OR 'devic disease':ab,ti OR 'disease devic':ab,ti OR 'devic syndrome':ab,ti OR 'syndrome devic':ab,ti OR 'devics syndrome':ab,ti OR 'syndrome devics':ab,ti OR 'devics neuromyelitis optica':ab,ti OR 'neuromyelitis optica devics':ab,ti OR 'neuromyelitis optica spectrum disorder':ab,ti OR 'neuromyelitis optica':ab,ti) AND nmo:ab,ti AND 'spectrum disorders':ab,ti	
#11	#1 AND #9	0
#12	(('multiple sclerosis':ab,ti OR 'sclerosis, multiple':ab,ti OR 'sclerosis, disseminated':ab,ti OR 'disseminated sclerosis':ab,ti OR ms:ab,ti) AND 'multiple sclerosis':ab,ti OR 'multiple sclerosis, acute fulminating':ab,ti)	139,268
#13	#1 AND #12	912
#14	('encephalomyelitis, acute disseminated':ab,ti OR 'acute disseminated encephalomyelitis':ab,ti OR 'acute disseminated encephalomyelitides':ab,ti OR 'disseminated encephalomyelitides, acute':ab,ti OR 'encephalomyelitides, acute disseminated':ab,ti OR 'disseminated encephalomyelitis, acute':ab,ti OR 'encephalomyelitis, postexanthem':ab,ti OR 'postexanthem encephalomyelitis':ab,ti OR 'postinfectious encephalomyelitis':ab,ti OR 'encephalomyelitis, postinfectious':ab,ti OR 'encephalitis, post-vaccinal':ab,ti OR 'encephalitis, post vaccinal':ab,ti OR 'encephalitis, postvaccinal':ab,ti OR 'postvaccinal encephalitis':ab,ti OR 'post-vaccinal encephalomyelitis':ab,ti OR 'encephalomyelitides, post-vaccinal':ab,ti OR 'encephalomyelitis, post-vaccinal':ab,ti OR 'post vaccinal encephalomyelitis':ab,ti OR 'post-vaccinal encephalomyelitides':ab,ti OR 'encephalitis, vaccination':ab,ti OR 'vaccination encephalitis':ab,ti OR 'post-vaccinal encephalitis':ab,ti OR 'encephalitides, post-vaccinal':ab,ti OR 'post vaccinal encephalitis':ab,ti OR 'post-vaccinal encephalitides':ab,ti)	3,528
#15	#1 AND #13	0

1.3. Cochrane library

Step	Search	Hits
#1	MeSH descriptor: [Gastrointestinal Microbiome] explode all trees	1644
#2	(Gastrointestinal Microbiome or Gut Microbiome or Gut Microbiomes or Gut Microflora or Gut Microbiota or Gut Microbiotas or Gastrointestinal Flora or Gut Flora or Gastrointestinal Microbiota or Gastrointestinal Microbiotas or Gastrointestinal Microbial Community or Gastrointestinal Microbial Communities or Gastrointestinal Microflora or Gastric Microbiome or Gastric Microbiomes or Intestinal Microbiome or Intestinal	10782

	Microbiomes or Intestinal Microbiota or Intestinal Microbiotas or Intestinal Microflora or Intestinal Flora or Enteric Bacteria):ti,ab,kw	
#3	#1 or #2	10782
#4	MeSH descriptor: [Multiple Sclerosis] explode all trees	5201
#5	(Multiple Sclerosis or Sclerosis, Multiple or Sclerosis, Disseminated or Disseminated Sclerosis or MS (Multiple Sclerosis) or Multiple Sclerosis, Acute Fulminating):ti,ab,kw	12930
#6	#4 or #5	12930
#7	#3 and #6	47
#8	MeSH descriptor: [Anti-N-Methyl-D-Aspartate Receptor Encephalitis] explode all trees	3
#9	(Anti-N-Methyl-D-Aspartate Receptor Encephalitis or Anti N Methyl D Aspartate Receptor Encephalitis or Anti-N-Methyl-D-Aspartate Receptor Encephalitides or Encephalitides, Anti-N-Methyl-D-Aspartate Receptor or Encephalitis, Anti-N-Methyl-D-Aspartate Receptor or Anti-NMDA Receptor Encephalitis or Anti NMDA Receptor Encephalitis or Anti-NMDA Receptor Encephalitides or Encephalitides, Anti-NMDA Receptor or Encephalitis, Anti-NMDA Receptor or Receptor Encephalitides, Anti-NMDA or Receptor Encephalitis, Anti-NMDA or Anti-NMDAR Encephalitis or Anti NMDAR Encephalitis or Anti-NMDAR Encephalitides or Encephalitides, Anti-NMDAR or Encephalitis, Anti-NMDAR or Non-paraneoplastic Anti-N-Methyl-D-Aspartate Receptor Encephalitis or Non paraneoplastic Anti N Methyl D Aspartate Receptor Encephalitis or Non-paraneoplastic Anti-NMDA Receptor Encephalitis or Non paraneoplastic Anti NMDA Receptor Encephalitis or Non-paraneoplastic Anti-NMDAR Encephalitis or Anti-NMDAR Encephalitides, Non-paraneoplastic or Anti-NMDAR Encephalitis, Non-paraneoplastic or Encephalitides, Non-paraneoplastic Anti-NMDAR or Encephalitis, Non-paraneoplastic Anti-NMDAR or Non paraneoplastic Anti NMDAR Encephalitis or Non-paraneoplastic Anti-NMDAR Encephalitides or Paraneoplastic Anti-N-Methyl-D-Aspartate Receptor Encephalitis or Paraneoplastic Anti N Methyl D Aspartate Receptor Encephalitis or Paraneoplastic Anti-NMDA Receptor Encephalitis or Paraneoplastic Anti NMDA Receptor Encephalitis or Paraneoplastic Anti-NMDAR Encephalitis or Anti-NMDAR Encephalitides, Paraneoplastic or Anti-NMDAR Encephalitis, Paraneoplastic or Encephalitides, Paraneoplastic Anti-NMDAR or Encephalitis, Paraneoplastic Anti-NMDAR or Paraneoplastic Anti NMDAR Encephalitis or Paraneoplastic Anti-NMDAR Encephalitides):ti,ab,kw	25
#10	#8 or #9	25
#11	#3 and #10	0
#12	(anti-CASPR2 or CASPR2 or Anti-contactin associated protein-like 2 or anti-CNTNAP2 CASPR2 or anti-CNTNAP2):ti,ab,kw	7
#13	#3 and #1	0

#14	(anti-CASPR2 or CASPR2 or Anti-contactin associated protein-like 2 or anti-CNTNAP2 CASPR2 or anti-CNTNAP2anti-leucine-rich glioma-inactivated 1 or LGI1 or Anti-LGI1):ti,ab,kw	18
#15	#3 and #12	0
#16	MeSH descriptor: [Neuromyelitis Optica] explode all trees	89
#17	(Neuromyelitis Optica or NMO Spectrum Disorder or NMO Spectrum Disorders or Neuromyelitis Optica (NMO) Spectrum Disorder or Neuromyelitis Optica Spectrum Disorders or Devic Neuromyelitis Optica or Devic Neuromyelitis Opticas or Neuromyelitis Optica, Devic or Neuromyelitis Opticas, Devic or Devic's Disease or Devics Disease or Disease, Devic's or Devic Disease or Disease, Devic or Devic Syndrome or Syndrome, Devic or Devic's Syndrome or Devics Syndrome or Syndrome, Devic's or Devic's Neuromyelitis Optica or Devics Neuromyelitis Optica or Neuromyelitis Optica, Devic's or Neuromyelitis Optica Spectrum Disorder or Neuromyelitis Optica (NMO) Spectrum Disorders):ti,ab,kw	23666
#18	#16 or #17	23666
#19	#3 and #18	99
#20	MeSH descriptor: [Myasthenia Gravis] explode all trees	341
#21	(Myasthenia Gravis or Myasthenia Gravis, Ocular or Ocular Myasthenia Gravis or Myasthenia Gravis, Generalized or Generalized Myasthenia Gravis or Muscle-Specific Receptor Tyrosine Kinase Myasthenia Gravis or Muscle Specific Receptor Tyrosine Kinase Myasthenia Gravis or Muscle-Specific Tyrosine Kinase Antibody Positive Myasthenia Gravis or Muscle Specific Tyrosine Kinase Antibody Positive Myasthenia Gravis or MuSK MG or MuSK Myasthenia Gravis or Myasthenia Gravis, MuSK or Anti-MuSK Myasthenia Gravis or Anti MuSK Myasthenia Gravis or Myasthenia Gravis, Anti-MuSK):ti,ab,kw	913
#22	#20 or #21	929
#23	#3 and #22	1
#24	MeSH descriptor: [Encephalomyelitis, Acute Disseminated] 4 tree(s) exploded	5
#25	(Encephalomyelitis, Acute Disseminated or Acute Disseminated Encephalomyelitis or Acute Disseminated Encephalomyelitides or Disseminated Encephalomyelitides, Acute or Encephalomyelitides, Acute Disseminated or Disseminated Encephalomyelitis, Acute or Encephalomyelitis, Postexanthem or Postexanthem Encephalomyelitis or Postinfectious Encephalomyelitis or Encephalomyelitis, Postinfectious or Encephalitis, Post-Vaccinal or Encephalitis, Post Vaccinal or Encephalitis, Postvaccinal or Postvaccinal Encephalitis or Post-Vaccinal Encephalomyelitis or Encephalomyelitides, Post-Vaccinal or Encephalomyelitis, Post-Vaccinal or Post Vaccinal Encephalomyelitis or Post-Vaccinal Encephalomyelitides or Encephalitis, Vaccination or Vaccination Encephalitis or Post-Vaccinal Encephalitis or Encephalitides, Post-Vaccinal or Post Vaccinal	365

	Encephalitis or Post-Vaccinal Encephalitides):ti,ab,kw	
#26	#24 or #25	365
#27	#3 and #26	0

1.4. Web of science

Step	Search	Hits
1	TS= ((Gastrointestinal Microbiome or Gut Microbiome or Gut Microbiomes or Gut Microflora or Gut Microbiota or Gut Microbiotas or Gastrointestinal Flora or Gut Flora or Gastrointestinal Microbiota or Gastrointestinal Microbiotas or Gastrointestinal Microbial Community or Gastrointestinal Microbial Communities or Gastrointestinal Microflora or Gastric Microbiome or Gastric Microbiomes or Intestinal Microbiome or Intestinal Microbiomes or Intestinal Microbiota or Intestinal Microbiotas or Intestinal Microflora or Intestinal Flora or Enteric Bacteria))	138870
2	TS= ((Anti-N-Methyl-D-Aspartate Receptor Encephalitis or Anti N Methyl D Aspartate Receptor Encephalitis or Anti-N-Methyl-D-Aspartate Receptor Encephalitides or Encephalitides, Anti-N-Methyl-D-Aspartate Receptor or Encephalitis, Anti-N-Methyl-D-Aspartate Receptor or Anti-NMDA Receptor Encephalitis or Anti NMDA Receptor Encephalitis or Anti-NMDA Receptor Encephalitides or Encephalitides, Anti-NMDA Receptor or Encephalitis, Anti-NMDA Receptor or Receptor Encephalitides, Anti-NMDA or Receptor Encephalitis, Anti-NMDA or Anti-NMDAR Encephalitis or Anti NMDAR Encephalitis or Anti-NMDAR Encephalitides or Encephalitides, Anti-NMDAR or Encephalitis, Anti-NMDAR or Non-paraneoplastic Anti-N-Methyl-D-Aspartate Receptor Encephalitis or Non paraneoplastic Anti N Methyl D Aspartate Receptor Encephalitis or Non-paraneoplastic Anti-NMDA Receptor Encephalitis or Non paraneoplastic Anti NMDA Receptor Encephalitis or Non-paraneoplastic Anti-NMDAR Encephalitis or Anti-NMDAR Encephalitides, Non-paraneoplastic or Anti-NMDAR Encephalitis, Non-paraneoplastic or Encephalitides, Non-paraneoplastic Anti-NMDAR or Encephalitis, Non-paraneoplastic Anti-NMDAR or Non paraneoplastic Anti NMDAR Encephalitis or Non-paraneoplastic Anti-NMDAR Encephalitides or Paraneoplastic Anti-N-Methyl-D-Aspartate Receptor Encephalitis or Paraneoplastic Anti N Methyl D Aspartate Receptor Encephalitis or Paraneoplastic Anti-NMDA Receptor Encephalitis or Paraneoplastic Anti NMDA Receptor Encephalitis or Paraneoplastic Anti-NMDAR Encephalitis or Anti-NMDAR Encephalitides, Paraneoplastic or Anti-NMDAR Encephalitis, Paraneoplastic or Encephalitides, Paraneoplastic Anti-NMDAR or Encephalitis, Paraneoplastic Anti-NMDAR or Paraneoplastic Anti NMDAR Encephalitis or Paraneoplastic Anti-NMDAR Encephalitides))	2782
3	#2 AND #1	8

4	(TS= ((anti-CASPR2 or CASPR2 or Anti-contactin associated protein-like 2 or anti-CNTNAP2 CASPR2 or anti-CNTNAP2))) AND TS= ((Encephalitis or Encephalitides))	409
5	#4 AND #1	0
6	(TS= ((anti-leucine-rich glioma-inactivated 1 or LGI1 or Anti-LGI1))) AND TS=((Encephalitis or Encephalitides))	808
7	#6 AND #1	1
8	TS=((Myasthenia Gravis or Myasthenia Gravis, Ocular or Ocular Myasthenia Gravis or Myasthenia Gravis, Generalized or Generalized Myasthenia Gravis or Muscle-Specific Receptor Tyrosine Kinase Myasthenia Gravis or Muscle Specific Receptor Tyrosine Kinase Myasthenia Gravis or Muscle-Specific Tyrosine Kinase Antibody Positive Myasthenia Gravis or Muscle Specific Tyrosine Kinase Antibody Positive Myasthenia Gravis or MuSK MG or MuSK Myasthenia Gravis or Myasthenia Gravis, MuSK or Anti-MuSK Myasthenia Gravis or Anti MuSK Myasthenia Gravis or Myasthenia Gravis, Anti-MuSK))	16859
9	#8 AND #1	48
10	TS= ((Neuromyelitis Optica or NMO Spectrum Disorder or NMO Spectrum Disorders or Neuromyelitis Optica (NMO) Spectrum Disorder or Neuromyelitis Optica Spectrum Disorders or Devic Neuromyelitis Optica or Devic Neuromyelitis Opticas or Neuromyelitis Optica, Devic or Neuromyelitis Opticas, Devic or Devic's Disease or Devics Disease or Disease, Devic's or Devic Disease or Disease, Devic or Devic Syndrome or Syndrome, Devic or Devic's Syndrome or Devics Syndrome or Syndrome, Devic's or Devic's Neuromyelitis Optica or Devics Neuromyelitis Optica or Neuromyelitis Optica, Devic's or Neuromyelitis Optica Spectrum Disorder or Neuromyelitis Optica (NMO) Spectrum Disorders))	9330
11	#10 AND #1	38
12	TS= ((Multiple Sclerosis or Sclerosis, Multiple or Sclerosis, Disseminated or Disseminated Sclerosis or MS (Multiple Sclerosis) or Multiple Sclerosis, Acute Fulminating))	151863
13	#12 AND #1	1102

14	TS= ((Encephalomyelitis, Acute Disseminated or Acute Disseminated Encephalomyelitis or Acute Disseminated Encephalomyelitides or Disseminated Encephalomyelitides, Acute or Encephalomyelitides, Acute Disseminated or Disseminated Encephalomyelitis, Acute or Encephalomyelitis, Postexanthem or Postexanthem Encephalomyelitis or Postinfectious Encephalomyelitis or Encephalomyelitis, Postinfectious or Encephalitis, Post-Vaccinal or Encephalitis, Post Vaccinal or Encephalitis, Postvaccinal or Postvaccinal Encephalitis or Post-Vaccinal Encephalomyelitis or Encephalomyelitides, Post-Vaccinal or Encephalomyelitis, Post-Vaccinal or Post Vaccinal Encephalomyelitis or Post-Vaccinal Encephalomyelitides or Encephalitis, Vaccination or Vaccination Encephalitis or Post-Vaccinal Encephalitis or Encephalitides, Post-Vaccinal or Post Vaccinal Encephalitis or Post-Vaccinal Encephalitides))	5809
15	#14 AND #1	7

Supplementary Table 2. Summary of Exclusion Criteria for Full-Text Articles

Number of Studies	Reason for Exclusion	Details
13	Clinical Trial Register Protocol	Studies lacking a control or comparator group, preventing meaningful data comparison for our meta-analysis.
4	Clinical Trial Register Protocol	Protocols or registrations for clinical trials that have not yet resulted in completed studies with analyzed data.
3	Non-English Language	Studies published in languages other than English, which were not included due to our study's language limitation.
7	Other Irrelevant Studies	Includes studies with data already covered in more comprehensive or recent publications (3 studies), and those lacking specific data on gut microbiota diversity or abundance required for our analysis (4 studies).

Supplementary Table 3. Detailed characteristics of the included studies

Disorder	Study	Country	Definition of disorder stage	Sample size	Mean age	Mean BMI (kg/m2)	% Female	% Patients on medication	Matching-variables
AIE	Gong et al. 2019[1]	China	The diagnostic criteria by Graus et al.[2]	P:30<br>C:12	P:31.90<br>C:30.40	P:24.70<br>C:21.61	P:40.00<br>C:25.00	0	Age, BMI, gender, eating habits, geographical space
AIE	Herken-2019[3]	Germany	The diagnostic criteria by Graus et al.	P:23<br>C:24	P: 34<br>C: 40	NA	P:91.30<br>C:91.67	21.74	Age, gender, living environment, diet
AIE	Chen-2020[4]	China	The diagnostic criteria by Graus et al.	P:40<br>C:54	P: 22.00<br>C: 23.00	P: 21.57<br>C: 20.43	P:55.00<br>C:55.56	0	Age, BMI, gender
AIE	Gong-2022[5]	China	The diagnostic criteria by Graus et al.	P:58<br>C:49	P: 34.4<br>C: 32.0	NA	P:62.07<br>C:63.27	0	Age, BMI, gender, diet, geographical space

AIE	Wei-2022[6]	China	The diagnostic criteria by Graus et al.	P:10<br>C:10	P:30.4<br>C:32.2	P:23.7<br>C:22.7	P: 40.0<br>C: 60.0	20	Age, BMI, gender
AIE	Ma-2020[7]	China	The diagnostic criteria by Graus et al.	P:15<br>C:25	P: 54.3<br>C: 55.3	P: 22.12<br>C: 23.01	P:40.0<br>C:36.0	0	Age, BMI, gender
MG	Moris-2018[8]	Spain	MGFA	P:10<br>C:10	P: 70.9<br>C: 70.6	NA	P:70.0<br>C:70.0	60.00	Age, gender
MG	Qiu-2018[9]	China	MGFA	P:53<br>C:50	P:43.6<br>C:46.2	P: 22.9<br>C: 23.1	P: 44.51<br>C: 38.0	0	Age, gender
MG	Zheng-2019[10]	China	NA	P:70<br>C:74	P: 46.12<br>C: 44.86	P:22.3<br>C:NA	P:55.71<br>C:59.46	44.3	Age, gender
MG	Liu-2021[11]	China	MGFA I	P:53<br>C:46	P: 6.64<br>C: 7.51	NA	P: 69.81<br>C: 45.65	0	Age, gender
MG	Totzeck-2021[12]	Germany	MGFA I-IIIB	P:41<br>C:12	P: 64.6<br>C: 51.4	P:27.7<br>C: 26.7	P: 41.46<br>C: 66.67	58.5	NA
MG	Ding-2023[13]	China	MGFA I-IIIB	P:11<br>C:11	P: 51.82<br>C: 52.91	P: 27.15<br>C: 25.94	P: 63.64<br>C: 45.45	0	Age, gender
MG	Zhao-2023[14]	China	NA	P:30<br>C:30	P: 59.47<br>C: NA	NA	NA	0	NA
NMOSD	Cree-2016[15]	USA	AQP4 seropositive	P:16<br>C:16	P: 47.9<br>C:53	P:26.04<br>C:23.83	P:75.0<br>C:43.75	93.75	Unaffected NMO-household controls

NMOSD	Gong-2019[16]	China	International consensus diagnostic criteria, AQP4 seropositive	P:84<br>C:54	P: 37.13<br>C: 38.61	P: 21.63<br>C: 21.91	P:95.3<br>C: 64.8	78.57	Age, BMI, gender
NMOSD	Zeng-2019[18]	China	International consensus diagnostic criteria, AQP4 seropositive	P:34<br>C:34	P: 31.21<br>C: 35.18	P: 21.11<br>C:21.72	P:91.18<br>C:61.76	79.41	Age, BMI, gender
NMOSD	Pandit-2020[19]	India	17 AQP4 seropositive, 22 AQP4 seronegative	P:39<br>C:37	P:35.78<br>C:33.7	P:23.75<br>C:23.4	P:33.33<br>C:59.46	82.05	Age, BMI
NMOSD	Shi-2020[20]	China	International consensus diagnostic criteria, AQP4 seropositive	P:20<br>C:20	P: 48.15<br>C:47.65	P:22.46<br>C:22.97	P: 90.0<br>C:45.0	65.0	Age, BMI, gender, dietary patter
NMOSD	Takewaki-2020[21]	Japan	International consensus diagnostic criteria, AQP4 seropositive	P:20<br>C:55	P: 43.1<br>C: 40.0	P:22.9<br>C:22.2	P:90.0<br>C:34.55	NA	Age, BMI

NMOSD	Zhang-2020 [22]	China	International consensus diagnostic criteria, 14AQP4 seropositive	P:22<br>C:28	P:47.76<br>C: 25.24	P: 21.29<br>C: 21.35	P:90.9<br>C:39.29	95.45	NA
MS	Cantarel-2015[23]	USA	2010 McDonald	P:7<br>C:8	P: 42<br>C: 38	NA	NA	71.43	age
MS	Miyake-2015[24]	Japan	2010 McDonald	P:20<br>C:40	P: 36.0<br>C: 28.53	NA	P: 70.0<br>C:52.5	62.5	NA
MS	Cree-2016[15]	USA	2010 McDonald	P:16<br>C:16	P: 54.27<br>C:54.13	P: 23.25<br>C:23.83	P: 50.0<br>C:43.75	31.25	Age, BMI, gender
MS	Chen-2016[25]	USA	2010 McDonald	P:31<br>C:36	P: 42.9<br>C: 40.3	P: 28.0<br>C: 27.8	P: 56.67<br>C: 54.55	64.52	age, gender
MS	Jangi-2016[26]	USA	2010 McDonald	P:60<br>C:43	P: 49.7<br>C: 42.2	P: 27.2<br>C: 26.4	P: 68.33<br>C: 86.05	53.33	NA
MS	Tremlett-2016[27]	USA	NA	P:15<br>C:9	P: 11.9<br>C: 13.8	NA	P: 53.33<br>C: 77.78	NA	age, gender
MS	Berer-2017[28]	Germany	2010 McDonald	P:34<br>C:34	P: 41.3<br>C: 41.3	P: 24<br>C: 25.8	P:76.47<br>C: 76.47	50.0	age, diet, twin
MS	Cekanaviciute-2017[29]	USA	2010 McDonald	P:71<br>C:71	NA	NA	NA	0	NA
MS	Swidsinski-2017[30]	Germany	NA	P:25<br>C:14	NA	NA	NA	NA	

MS	Forbes-2018[31]	Canada	2010 McDonald criteria	P:19<br>C:23	P:47.3<br>C:32.4	NA	P:73.68<br>C:52.17	NA	NA
MS	Tankou-2018[32]	USA	2010 McDonald criteria	P:9<br>C:13	P: 50<br>C: 35	P:31.1<br>C:25.8	P: 55.6<br>C:61.5	77.78	NA
MS	Kozhieva-2019[33]	Russian	2010McDonald criteria	P:15<br>C:15	P: 45<br>C: 23	P: 22<br>C: 24	P: 40<br>C: 44	0	NA
MS	Oezguen-2019[34]	Turkey	2010 McDonald criteria	P:13<br>C:14	P: 39.1<br>C: 37.8	NA	P: 61.54<br>C: 28.57	100.0	age, gender, diet
MS	Storm-Larsen-2019[35]	Norwegian	NA	P:34<br>C:165	P: 46<br>C: 47	P: 24<br>C: 25.8	P: 73.53<br>C: 63.03	0	diet
MS	Ventura-2019[36]	USA	2010 McDonald Criteria	P:45<br>C:44	P: 37.1<br>C: 31.8	NA	P: 76<br>C: 64	9.0	ethnicity
MS	Zeng-2019[18]	China	2017 McDonald	P:34<br>C:34	P:29<br>C: 35.18	P: 21.39<br>C:21.72	P:61.76<br>C: 61.76	38.2	
MS	Choileain-2020[37]	USA	NA	P:26<br>C:39	P: 42<br>C: 45	P: 29<br>C: 27	P: 84.62<br>C:69.23	0	age, gender, BMI
MS	Kishikawa-2020[38]	Japan	2010 McDonald criteria	P:26<br>C:77	P:44.98<br>C:30.22	NA	NA	100.0	NA

MS	Ling-2020[39]	China	2005 McDonald criteria	P:22<br>C:33	P: 35.0<br>C: 34.5	P: 24.18<br>C: 23.85	P: 63.64<br>C: 63.64	0	age, gender, BMI
MS	Reynders-2020[40]	Belgium	2010 McDonald criteria	P:98<br>C:120	P: 48.0<br>C: 49.0	P: 23.6<br>C: 23.7	P:60.2<br>C:61.7	≥24.5	age, gender, BMI
MS	Saresella-2020[41]	Italy	2017<br>McDonald criteria	P:38<br>C:38	P:47<br>C:48	NA	P: 52.63<br>C: 52.63	0	age, gender, diet, ethnicity
MS	Takewaki-2020[21]	Japan	2017<br>McDonald criteria	P:98<br>C:55	P:40.37<br>C: 40.0	P: 21.94<br>C: 22.2	P:71.43<br>C:34.55	83.7	Age, BMI
MS	Barone-2021[42]	Italy	NA	P:14<br>C:17	NA	P:49.43<br>C: NA	P:50<br>C:59.26	0	NA
MS	Cox-2021[43]	USA	2017<br>McDonald criteria	P:243<br>C:40	P: 50.84<br>C: 45.4	P: 27.34<br>C: 28	P:755.31<br>C: 70.0%	81.5	NA
MS	Elgendy-2021[44]	Egypt	2017 McDonald criteria	P:40<br>C:22	P: 31.4<br>C: 30.4	NA	P: 75.0<br>C: 77.27	0	age, gender
MS	Galluzzo-2021[45]	Italy	NA	P:15<br>C:15	P: 28–66<br>C: 21–69		P:11/15<br>C:7/15	0	diet
MS	Levi-2021[46]	Israel	McDonald criteria	P:129<br>C:58	P:38.3<br>C:45.8	P:24.1<br>C:25.9	P:72.09<br>C:50.0	NA	NA

MS	Mekky-2021[47]	Egyptian	2017 McDonald criteria	P:30<br>C:22	P:31.43<br>C: 32.3	NA	P: 56.67<br>C: 54.55	100.0	age, gender
MS	Mirza-2021[48]	Canada	2017 McDonald criteria	P:20<br>C:20	P:16.1<br>C:15.4	P:22.8<br>C:21.0	P:80<br>C:80	60.0	Age, gender, ethnicity
MS	Pellizoni-2021[49]	Brazil	Poser and colleagues criteria[50]	P:18<br>C:18	P: 46.06<br>C: 45.50	NA	P: 88.89<br>C: 88.89	100	age, gender
MS	Sterlin -2021[51]	France	McDonald	P:48<br>C:32	P: 36.67<br>C:35.09	P:23.69<br>C:22.61	P:66.67<br>C:62.5	0	age, gender
MS	Tremlett-2021[52]	Canada-USA	2017McDonald criteria	P:32<br>C:36	P: 16.5<br>C: 15.1	P: 22.8<br>C: 19.9	P:24/32<br>C: 21/36	72	age, gender
MS	Ascanelli-2022[53]	Italy	NA	P:17<br>C:17	NA	NA	NA	NA	Age, gender
MS	Bruijstens-2022[54]	Netherlands	International Pediatric MS Study Group criteria	P:26<br>C:24	P: 17.3<br>C: 10.6	NA	P: 65.4<br>C: 62.5	92	NA
MS	Cantoni-2022[55]	USA	2010 McDonald criteria	P:24<br>C:25	P: 40.2<br>C: 38.9	P: 27·3<br>C: 26·9	P:87.5<br>C:88.0	0	age, gender, BMI, ethnicity

MS	Zhou-2022[56]	USA, Europe, South America	McDonald criteria	P:500<br>C:500	P: 48.9<br>C: 50.6	P: 25.4<br>C: 26.9	P: 69.4<br>C: 34.9	64.2	diet, ethnicity
MS	Moles-2022[57]	Spain	NA	P:20<br>C:20	P: 47.1<br>C: 49.2	NA	P: 80.0<br>C: 15.0	80.0	diet, ethnicity
MS	Navarro-López-2022[58]	Spain	2017 McDonald criteria	P:15<br>C:15	P:38.15<br>C: NA	NA	P 86.67<br>C:	100.0	Age, diet
MS	Troci-2022[59]	Germany	2017<br>McDonald criteria	P:54<br>C:36	P:42.94<br>C:47	P: 24.66<br>C:25	P:83.33<br>C:59.26	NA	Age, gender
MS	Elsayed-2023[60]	USA	NA	P:117<br>C:26	P:50.0<br>C:42.30	P: 29.8<br>C:27.64	P:68.4<br>C:69.2	70.4	NA
MS	Nitzan-2023[61]	Israel	2017<br>McDonald criteria	P:57<br>C:43	P: 33.6<br>C:38.1	P: 25.4<br>C:24.7	P:70.2<br>C:58.1	0	Age, gender, BMI,smoking, diet, ethnicity
MS	Thirion-2023[62]	Denmark	McDonald	P:148<br>C:148	P: 36<br>C: 36	P:24<br>C:23	P:66.22<br>C:66.22	64.0	age, gender
MS	Vacaras-2023[63]	Romania	2017<br>McDonald criteria	P:50<br>C:21	P: 30.5<br>C: 28	NA	P:62.0<br>C:61.9	0	age, gender
MS	Schoeps-2024[64]	USA	2010 McDonald criteria	P:35<br>C:35	P: 11.9<br>C: 13.8	NA	P: 27/35<br>C: 26/35	54.0	Age, gender, ethnicity

NBD	Oezguen-2019[34]	Turkey	fulfill the diagnostic criteria for BD 1990[65]	P:13<br>C:14	P: 42.1<br>C: 37.8	NA	P: 38.46<br>C: 28.57	100.0	age, gender, diet
DON	Liu-2023[66]	China	NA	P:54<br>C:41	P: 38.70<br>C: 38.56	P: 23.53<br>C: 23.39	P:59.25<br>C:65.85	0	Age, gender, BMI

P: patients with neurological autoimmune diseases; C: healthy controls; AIE: autoimmune encephalitis; MG: myasthenia gravis; NMOSD: optic neuromyelitis optica spectrum disorders; MS: multiple sclerosis; NBD: neuro-behcet's disease; BD: behcet's disease; DON: demyelinating optic neuritis; CI, confidence interval. NA, not applicable.

Supplementary Table 4. Quality assessment of the included studies using the Newcastle-Ottawa Scale (NOS).

The quality of the included research was assessed using the Newcastle-Ottawa Quality Assessment Scale (NOS). Those equal to or less than 5 were deemed indicative of low quality, while scores of 6 or 7 signified moderate quality, and scores of 8 or 9 indicated high quality. XL Deng and X Gong independently conducted assessments to determine the quality of the research involved in the analysis. In cases of score disagreement, the two reviewers examined the articles together to reach a consensus.

Case-control studies	Selection				Comparability	Exposure			Total
	Case definition	Representativeness of the case	Selection of controls	Definition of controls	Based on design and analysis	Assessment of exposure	Same measurement for case and controls	Non-respondents	0-9
Gong-2019	1	0	1	1	1	1	1	0	6
Herken-2019	1	0	1	1	1	1	1	0	6
Chen-2020	1	0	1	1	1	1	1	0	6
Gong-2022	1	0	1	1	1	1	1	0	6
Wei-2022	0	0	1	1	1	1	1	0	5
Ma-2020	1	0	1	1	1	1	1	0	6
Moris-2018	0	0	0	1	1	1	1	1	5
Qiu-2018	1	0	1	1	1	1	1	1	7
Zheng-2019	0	0	1	1	1	1	1	1	6
Liu-2021	1	0	1	1	1	1	1	1	7
Totzeck-2021	1	0	1	1	0	1	1	1	6
Ding-2023	1	0	1	1	1	1	1	1	7
Zhao-2023	0	0	0	1	0	1	1	1	4
Cree-2016	1	0	1	1	1	0	1	1	6

Gong-2019	1	1	1	1	1	1	1	0	7
Zeng-2019	1	0	1	1	1	1	1	0	6
Pandit-2020	1	0	1	1	1	1	1	1	7
Shi-2020	1	0	1	1	1	1	1	0	6
Takewaki-2020	1	0	1	1	1	1	1	1	7
Zhang-2020	1	0	1	1	0	0	1	1	5
Cantarel-2015	1	0	0	1	1	1	1	0	5
Miyake-2015	1	0	1	0	1	1	1	0	5
Chen-2016	1	0	0	1	1	1	1	0	5
Jangi-2016	1	0	1	1	1	1	1	1	7
Tremlett-2016	0	0	0	1	1	1	1	1	5
Berer-2017	1	0	1	1	1	1	1	1	7
Cekanaviciute-2017	1	0	1	1	1	1	1	1	7
Swidsinsk-2017	0	0	1	1	0	1	1	0	4
Forbes-2018	1	0	1	1	0	1	1	0	5
Tankou-2018	1	0	1	1	1	1	1	1	7
Kozhieva-2019	1	0	0	1	0	1	1	1	5
Oezguen-2019	1	0	1	1	1	1	1	1	7
Storm-Larsen-2019	0	0	1	1	1	1	1	1	6
Ventura-2019	1	0	1	1	1	1	1	1	7
Choileain-2020	0	0	1	1	1	1	1	1	6
Kishikawa-2020	1	0	0	1	0	1	1	1	5
Ling-2020	1	0	1	1	1	1	1	1	7
Reynders-2020	1	0	1	1	1	1	1	0	6
Saresella-2020	1	0	1	1	1	1	1	0	6

Barone-2021	0	0	0	1	1	1	1	1	5
Cox-2021	1	0	1	1	1	1	1	1	7
Elgendy-2021	1	0	1	1	1	1	1	0	6
Galluzzo-2021	0	0	0	1	1	1	1	1	5
Levi-2021	1	0	1	1	1	1	1	1	7
Mekky-2021	1	0	1	0	1	1	1	0	5
Mirza-2021	1	0	1	1	1	1	1	1	7
Pellizoni-2021	1	0	0	1	1	1	1	1	6
Sterlin-2021	1	0	1	1	1	1	1	1	7
Tremlett-2021	1	0	1	1	1	1	1	0	6
Ascanelli-2022	0	0	0	1	1	1	1	0	4
Bruijstens-2022	1	0	1	1	0	1	1	1	6
Cantoni-2022	1	0	1	1	2	1	1	1	8
Zhou-2022	1	1	1	1	1	1	1	1	8
Moles-2022	0	0	1	1	1	1	1	1	6
Navarro-López-2022	1	0	1	1	1	1	1	0	6
Troci-2022	1	0	0	1	1	1	1	1	6
Elsayed-2023	0	0	1	1	0	1	1	1	5
Nitzan-2023	1	0	1	1	1	1	1	1	7
Thirion-2023	1	0	1	1	2	1	1	0	7
Vacaras-2023	1	0	0	1	1	1	1	1	6
Schoeps-2024	1	0	1	1	1	1	1	0	6
Liu-2023	1	0	1	1	1	1	1	0	6

Supplementary Table 5. Methodology of stool processing of the included studies

Stool processing methods and compositional analyses also varied significantly. Sequencing of 16S ribosomal RNA (16S rRNA) was the most widely applied method, used with 54 studies, followed by shotgun metagenomics with 12 studies (5 of which exclusively used only the metagenomics), real-time quantitative polymerase chain reaction with 2 studies, and fluorescence in situ hybridization in one study.

Study	Sequencing	Collection & handling by participant	Long-term storage	DNA extraction method
Gong-2019	16S rRNA V4	Fecal samples were delivered to West China Hospital within 2 h and and stored at 80°C	At -80°C	CTAB/SDS method
Herken-2019	16S rRNA V1-V2	Fecal samples were in standard stool collection tubes and shipped immediately (within 24 hours) at room temperature and were stored at −80°C until processing	At -80°C	QIAcube and the QIAamp DNA stool kit (Qiagen) and a prior beat-beating step
Chen-2020	16S rRNA V4	Fecal samples were collected and immediately positioned in standard sterile anaerobic collection tubes	At -80°C	QIAamp DNA Stool Mini Kit (Qiagen, Germany) according to the manufacturer's instructions.
Gong-2022	16S rRNA V3-V4	Participants discharged their feces into the sterile potty, took the middle part of the feces, instantly put them on ice, and stoerd at −80°C within 1–2 h after sample collection	At -80°C	E.Z.N.A.® Soil DNA Kit (Omega Bio-Tek, Norcross, GA, USA) according to the manufacturer's instructions
Wei-2022	16S rRNA V3-V4	Fecal samples were gathered into sterile drying tubes within minutes to hours of deposition rozen at -80°C immediately or at 4°C until they were transferred to -80°C (typically within 6 h)	At -80°C	QIAamp DNA Stool Mini Kit (QIAGEN, Hilden, Germany) according to the manufacturer's instructions

Ma-2020	16S rRNA V4	Stool samples were collected on the morning after admission and before immunotherapy.	At -80℃	QIAamp DNA Stool Mini Kit (Qiagen, Germany)
Moris-2018	16S rRNA V3	The volunteers provided a fresh fecal sample that was immediately frozen (-20 °C) until analyses	At -20℃	QIAamp DNA stool kit (Qiagen, GmbH, Hilden, Germany)
Qiu-2018	16S rRNA V4	Fecal samples were in the scoop attached to the screw-capped container. All collected samples were preserved at 4◦C during transportation	At -80℃	DNA Isolation Kit (MoBio, Carlsbad, CA, United States) according to the manufacturer's protocol
Zheng-2019	16S rRNA V3-V4	Fresh stool samples were collected from each participant and immediately frozen at -80 °C until further analysis	At -80℃	QIAampDNA Stool Mini Kit (Qiagen, Hilden, Germany)
Liu-2021	Metagenomics	Approximately 2 g of a fresh fecal sample was collected in a Fecal collection tube (OMR-200 DNA Genotek) and stored at room temperature until DNA extraction (21–28 days)	At room temperature	TruSeq DNA Nano Reference Guide (1000000040135) and Hiseq 2500 System Guide (15035786) from Illumina
Totzeck-2021	16S rRNA V3-V4	Fresh faecal samples were collected from participants in the morning. Faeces were transferred into provided collection tubes using an enclosed spoon and returned to study staff who transported them to the Institute of Medical Microbiology at 4°C within 12 h of specimen collection	At -80℃	QIAmp Fast DNA Stool Mini kit (Qiagen), following the manufacturer's instructions.
Ding-2023	16S rRNA V3-V4	Fecal samples were collected immediately, transported to the laboratory, and promptly frozen at −80°C	At -80℃	OMEGA Soil DNA Kit (M5635-02) (Omega Bio-Tek, Norcross, GA, United States)

Zhao-2023	16S rRNA V3-V4	Fecal samples were collected once the healthy control group was enrolled	At -20℃	DNA Extraction Kit (MP biomedical, Santa Ana, CA, United States) according to the manufacturer’s instructions
Cree-2016	16S rRNA	NA	NA	MoBio PowerMag Soil DNA Isolation Kit as per the vendor’s protocol
Gong-2019	16S rRNA V3-V4	Fecal sample aliquots from the participants were frozen at -80°C immediately after collection	At -80℃	QIAamp DNA stool Mini Kit (Qiagen, Germany), according to the manufacturer’s instructions.
Zeng-2019	16S rRNA V3-V4	Stool samples were collected at one study center and stored at −80 °C immediately	At -80℃	QIAamp DNA Stool Mini Kit (Qiagen, Germany), following the manufacturer's instructions.
Pandit-2020	16S rRNA V4	Stool samples were collected in containers, and were delivered on the same day of collection over a median period of 3 hours (range: 1–5 hours).Stool samples were then immediately frozen at -80℃	At -80℃	QIAamp® DNA Stool extraction kit as per the manufacturer’sinstruction
Shi-2020	16S rRNA V3-V4	A fresh stool sample was obtained from each participant immediately after defecation using a sterile stool collector and stored at −80 °C	At -80℃	QIAamp Fast DNA Stool Mini Kit (Qiagen, Hilden, Germany) according to the manufacturer's protocol
Takewaki-2020	16S rRNA V1-V2	Freshly collected fecal samples were transported at 4 °C to the laboratory in a plastic bag containing a disposable oxygen-absorbing and carbon dioxidegenerating agentIn the laboratory, the fecal samples were suspended in phosphate-buffered saline containing 20% glycerol, immediately frozen using liquid nitrogen, and stored at −80 °C	At -80℃	Enzymatic lysis methods

Zhang-2020	16S rRNA V3-V4	NA	NA	Standard commercial kits according to the standard protocol (G-BIO Technologies, Hangzhou, China)
Cantarel-2015	16S rRNA	Samples were shipped overnight on ice packs to the processing facility, where they were immediately stored at−80 °C	At -80℃	UltraClean Fecal DNA Isolation Kit (MoBio, Carlsbad, CA)
Miyake-2015	16S rRNA V1-V2	Freshly collected fecal samples were transported at 4°C to the laboratory in a plastic bag containing a disposable oxygen-absorbing and carbon dioxide-generating agent in which anaerobes sensitive to oxygen can survive. In the laboratory, the fecal samples were suspended in phosphate-buffered saline containing 20% glycerol, immediately frozen using liquid nitrogen, and stored at -80°C	At -80℃	enzymatic lysis methods
Chen-2016	16S rRNA V3–V5	Samples were frozen at −70 °C within 24 hours of receipt	At -70℃	MoBio PowerSoil Kit (MoBio Laboratories, Carlsbad, CA, USA) as per the manufacturer's instruction
Jangi-2016	16S rRNA V3-V5, V4	Collection containers were then placed in boxes with provided ice packs for immediate shipment to our laboratory via overnight delivery at a maintained temperature of 0 °C. On receipt of samples, they were frozen at 80 °C	At −80 °C	PowerSoil DNA Isolation kit (MO BIO Laboratories, Carlsbad, CA, USA) with the Human Microbiome Project modifications to the manufacturer's protocol
Tremlett-2016	16S rRNA V4	Participants were asked to collect and ship overnight (on ice) a sample of the child's first stool of the day to UCSF where it was stored at −80 °C	At -80℃	PowerSoil® DNA Isolation Kit (MO BIO Laboratories, Inc, Carlsbad, CA)

Berer-2017	16S rRNA V3-V5	Fecal samples were directly collected in hospital or were taken at home, stored at −20 °C, and transferred to the hospital in cooling bags. Finally, all samples were stored at −80 °C	At -80°C	NA
Cekanaviciute-2017	16S rRNA V4	Samples were collected using culture swabs (BD #220135) and stored at -80C	At -80°C	MoBio Power Fecal DNA extraction kit (MoBio #12830)
Swidsinsk-2017	fluorescence in situ hybridization	NA	NA	NA
Forbes-2018	16S rRNA V4	Each participant self-collected two stool specimens approximately 2 months apart. The stool samples were kept refrigerated at 4 °C until transport. The stool was transported to the laboratory on ice and stored at − 80 °C	At -80°C	ZR-96 Fecal DNA Kit (Zymo Research, Irvine, CA) following a validated protocol
Tankou-2018	16S rRNA V4	Subjects collected two samples at each time point produced at any time of day with no specific dietary restrictions. Collection containers were then placed in boxes with provided ice packsfor immediate shipment to laboratory via overnight delivery at a maintained temperature of 0°C. On receipt of samples, they were frozen at −80°C	At -80°C	MoBio PowerLyzer PowerSoil Kit
Kozhieva-2019	16S rRNA V3-V4	Faecal samples were collected into 10 ml sterile faecal specimen containers and stored frozen at approximately −20 °C. Samples were transferred to the laboratory within 1 week of collection and stored at − 80 °C until used for DNA extraction	At -80°C	MetaHIT protocol

Oezguen-2019	16S rRNA V3-V5	All samples were frozen and kept at -80°C	At -80°C	PowerSoil isolation kit (MO BIO Laboratories, Carlsbad, California, USA)
Storm-Larsen-2019	16S rRNA V3-V4	Stool samples were collected by the patient at home in special tubes with preservatives (PSP tubes, Stratec), shipped to the central study laboratory and stored at –80°C	At -80°C	PSP Spin Stool DNA Kit (Stratec Molecular GmbH)
Ventura-2019	16S rRNA V4	Subjects were provided with stool collection containers as well as coolers with ice packs. Once the sample was collected and placed in the coolers, the sample was either picked up by courier or brought into the clinic within 24 hours of production, then were stored at −80 °C	At -80°C	PowerSoil DNA Isolation Kit (MOBIO, West Carlsbad CA), according to the manufacturer's protocol
Choileain-2020	16S rRNA V4	Donors collected stool at home using a provided kit. Samples were collected at room temperature in RNAlater® solution (Ambion, cat. AM7021) and shipped overnight. Samples were then aliquoted and frozen at 70 C	At -70 °C	Mobio PowerSoil DNA isolation kit (cat. 12888–100)
Kishikawa-2020	Metagenomics	Fecal samples had been immediately frozen after production in an insulated container for storage at −20°C and subsequently stored at −80°C within 24 h after production	At -80°C	NA
Ling-2020	16S rRNA V3-V4	Approximately 2 g of a fresh fecal sample was collected in a sterile plastic cup, and stored at −80°C after preparation within 15 min	At -80°C	QIAamp® DNA Stool Mini Kit (QIAGEN, Hilden, Germany) according to the manufacturer's instructions

Reynders-2020	16S rRNA V4	Samples were stored at 20°C immediately after sampling, in the participants' home freezer or at the NMSC, and transferred on dry ice to 80°C within 48 h	At -80°C	Adapted Mobio PowerMicrobiome DNA/RNA isolation Kit-based protocol.
Saresella-2020	16S rRNA V1-V3	NA	NA	DNeasy Blood and Tissue kit (QIAGEN) with the modifications
Barone-2021	16S rRNA V3-V4	NA	NA	NA
Cox-2021	16S rRNA V4	Study subjects collected a stool sample at home, then shipped samples overnight on icepacks to the laboratory, and samples were frozen at 80C upon receipt	At -80°C	DNAeasy PowerLyzer Microbiome DNA extraction kit (QIAGEN, Hilden, Germany)
Elgendy-2021	qPCR	Stool samples collected from patients before initiation of therapy and stored immediately after collection in screw cap sterile containers at -70°C	At -70°C	QIAamp® DNA Stool mini kit (Qiagen GmbH, Germany. Cat. No.12830-50) according to the manufacturer's protocol
Galluzzo-2021	16S rRNA V3-V4	Faecal samples were collected in tubes containing RNA Later and maintained at 4 °C within 24 h of receipt	NA	Metagenomic DNA was extracted using the QIAmp DNA Stool Mini Kit (Qiagen, West Sussex, UK), following the manufacturer's instructions
Levi-2021	Metagenomics	Collected samples were immediately stored in a home freezer (−20°C), and transferred in a provided cooler to our facilities where it was stored at −80°C (−20°C for OMNIIgene-GUT kits) until DNA extraction	At -80°C	PowerMag Soil DNA isolation kit (MoBio) optimized for Tecan automated platform.

Mekky-2021	qPCR	Stool specimens were collected, kept in the freezer upon defecation at home, and within the same day delivered to our laboratory frozen, where aliquots of each specimen were frozen at 80 ◦C	At -80°C	ISOLATE Fecal DNA Kit (Bioline, UK) according to the manufacturers' instructions
Mirza-2021	Metagenomics	Stool was collected and shipped on ice, then stored at −80°C	At -80°C	Zymo Quick-DNA Fecal/Soil Microbe Miniprep Kit (D6010).
Pellizoni-2021	16S rRNA V3-V4	stool samples were requested and delivered within five days	At -80°C	QIAamp DNA Stool Mini Kit (QIAGEN, Hilden, Germany), according to the manufacturer's instructions
Sterlin-2021	16S rRNA V3-V4	Stool samples were collected in a container including a reagent for the generation of an O2-depleted and CO2-enriched atmosphere (Anaerocult band, Mikrobiologie), aliquoted in an anaerobic atmosphere, and stored at −80°C.	At -80°C	Genomic DNA was extracted from whole stool samples as previously described
Tremlett-2021	16S rRNA V3-V4	The same collection kits were used for all participants, with stool shipped on ice before -80°C storage in the central laboratories	At -80°C	Zymo Quick-DNATM Fecal/Soil Microbe Miniprep Kit (D6010)
Ascanelli-2022	16S rRNA V3-V4	NA	NA	Repeated bead-beating plus column method
Bruijstens-2022	16S rRNA V3-V4	These samples were compiled at home using gut microbiome DNA collection kits from OMNIgene•GUT (DNA Genotek, Ottawa, Ontario, Canada) with stabilizing agent included and were sent through regular mail to the Erasmus University Medical Center	At -80°C	Microbial DNA was extracted from the stool samples and 16S ribosomal RNA gene sequencing of the V3 and V4 variable regions was performed on an Illumina MiSeq sequencer

Cantoni-2022	16S rRNA V1-V3	Stools were self-collected and placed on frozen gel packs and shipped overnight to the research laboratory. Upon receipt, stools were immediately stored at -80 °C	At -80°C	MOBIO PowerSoil DNA Extraction kit
Zhou-2022	16S rRNA V4	Participants were provided with a stool sample collection kit and instructed to obtain two consecutive stool samples in the privacy of their own homes. Each stool sample time point included 3 collection vials - a Q-tip (Q, dry), a snap frozen vial (S, wet), and a vial filled with Luria-Bertani broth and 30% glycerol. Participants were instructed to freeze the samples for at least 12 h and ship them frozen with the ice pack included in the kit. Samples were returned to each site via overnight shipping in a thermal envelope.	At -80°C	QIAamp PowerFecal DNA Kit (ref 12830-50) or on a QIAcube platform according to the protocols generated by the manufacturer (QIAGEN)
Moles-2022	16S rRNA	Participants were instructed about stool collection and transport. Fecal samples were immediately frozen at -20°C and were subsequently transported to the hospital protected with a cold accumulator. Once in the center, samples were stored at -80°C	At -80°C	QIAamp DNA Stool Mini Kit (Qiagen, Germany)
Navarro-López-2022	16S rRNA V3-V4	Stool samples were obtained from all the participants and were immediately frozen and stored at −80 ◦C	At -80°C	MagnaPure Compact System (Roche Life Science, Mannheim, Germany)

Troci-2022	16S rRNA V1-V2	NA	NA	QIAamp DNA fast stool mini kit automated on the QIAcube (Qiagen, Hilden, Germany)
Elsayed-2023	16S rRNA V4	The fecal samples were collected in a self-collection fecal sample kitwere returned in a boxed frozen cold pack to laboratory where they were divided into aliquots	At -80°C	PowerLyzer PowerSoil DNA Isolation Kit (MoBio Laboratories, Inc., Carlsbad, CA) by following the manufacturer's protocol
Nitzan-2023	16S rRNA V3-V4	Fecal samples were obtained using a stool preservative tube (Norgen Biotek, Thorold, ON, Canada), frozen immediately at arrival at clinic, and kept at −80°C	At -80°C	QIAamp® PowerFecal® Pro DNA kit (Qiagen, Tegelen, The Netherlands), according to manufacturer's protocol.
Thirion-2023	Metagenomics	Stools were collected according to International Human Microbiome Standards (IHMS) guidelines (SOP 03 V1) in kits at home and immediately stored at −20 °C until they were transported on dry ice and frozen 4–24 h later at −80°C in plastic tubes	At -80°C	aliquot of fecal samples was performed following IHMS SOP P7 V2
Vacaras-2023	16S rRNA<br>V1-V3 /V3-V4	fecal samples were collected using special stool containers, and were stored at minus 20 degrees Celsius and then shipped to the laboratory for the DNA extraction	At -20°C	NA
Schoeps-2024	16S rRNA V4	The participant's first stool of the day was collected by a parent and shipped overnight on ice to the University of California, San Francisco and stored at -80°C	At-80°C	Modified cetyltrimethylammonium bromide (CTAB) buffer based protocol

Liu-2023	16S rRNA V3-V4	All fresh fecal samples obtained from the participants within a sterile box were immediately transported (dry ice was used during transportation) to the laboratory and stored at −80 °C	At -80°C	DNA extraction kit (QIAamp PowerFecal Pro-DNA Kit (50), 51,804), according to the manufacturer's protocol

Supplementary Table 6. Results of subgroup analyses of different study regions

The subgroup analysis of geographical distribution of study populations was performed via comparing countries in the East and West. Eastern countries comprise East and South Asian nations, while Western countries encompass North America, Europe, and the Middle East.

**Alpha diversity metrics**	**Region of study**	**Pooled results**		**Heterogeneity**	
		**SMD (95% CI)**	**P values**	**I^2**	**P values**
Observed species	East	-0.15 (-0.41 to 0.11)	0.27	71	<0.01
	West	-0.21 (-0.77 to 0.35)	0.46	85	<0.01
Chao1	East	-0.23 (-0.47 to 0.02)	0.07	79	<0.01
	West	-0.36 (-0.83 to 0.12)	0.02	46	<0.01
ACE	East	-0.21 (-0.63 to 0.21)	0.32	83	<0.01
	West	-0.56 (-1.21 to 0.09)	0.09	66	0.05
Shannon index	East	-0.27 (-0.47 to -0.07)	0.01	72	<0.01
	West	0.08 (-0.10 to 0.27)	0.38	69	<0.01
Simpson index	East	0.02 (-0.26 to 0.31)	0.87	80	<0.01
	West	0.15 (-0.19 to 0.49)	0.38	60	0.01

SMD, standardised mean difference; CI, confidence interval

Supplementary Table 7. Results of subgroup analyses of studies with patients on treatment or treatment naive studies

The subgroup analysis of use of immunotherapy was performed via comparing patients receiving treatment and those without it. Studies where at least 80% of patients received immunotherapy were classified as having treated patients.

**Alpha diversity metrics**	**Immunotherapy**	**Pooled results**		**Heterogeneity**	
		**SMD (95% CI)**	**P values**	**I^2(%)**	**P values**
Observed species	On treatment*	0.12 (-0.21 to 0.45)	0.47	58	0.04
	Treatment naive	-0.31 (-0.62 to 0.00)	0.05	80	<0.01
Chao1	On treatment*	-0.11 (-0.41 to 0.20)	0.49	65	<0.01
	Treatment naive	-0.34 (-0.58 to -0.09)	<0.01	75	<0.01
ACE	On treatment*	-0.12 (-0.82 to 0.58)	0.73	-	-
	Treatment naive	-0.32 (-0.69 to 0.06)	0.10	82	<0.01
Shannon index	On treatment*	0.14 (-0.17 to 0.45)	0.38	74	<0.01
	Treatment naive	-0.25 (-0.41 to -0.10)	<0.01	68	<0.01
Simpson index	On treatment*	0.24 (-0.01 to 0.50)	0.06	44	0.06
	Treatment naive	-0.07 (-0.38 to 0.24)	0.66	81	<0.01

SMD, standardised mean difference; CI, confidence interval. * Studies with patients on treatment are those in which 80% or more of patients are receiving immunotherapy.

Supplementary Table 8. Results of sensitivity analyses by excluding studies with low quality (Newcastle-Ottawa Scale ≤5)

Alpha diversity metrics	Pooled results		Heterogeneity	
	**SMD (95% CI)**	***P* values**	**I^2**	***P* values**
Observed species	-0.22 (-0.48 to 0.04)	0.10	77	<0.01
Chao1	-0.31 (-0.51 to -0.11)	<0.01	73	<0.01
ACE	-0.31 (-0.72 to 0.10)	0.13	84	<0.01
Shannon index	-0.08 (-0.24 to 0.07)	0.31	76	<0.01
Simpson index	0.12 (-0.13 to 0.38)	0.33	77	<0.01

SMD, standardised mean difference; CI, confidence interval

Supplementary Table 9. Results of sensitivity analyses by excluding studies without matching any variables

Alpha diversity metrics	Pooled results		Heterogeneity	
	**SMD (95% CI)**	***P* values**	**I^2**	***P* values**
Observed species	-0.13 (-0.35 to 0.09)	0.24	69	<0.01
Chao1	-0.26 (-0.45 to -0.07)	<0.01	69	<0.01
ACE	-0.21 (-0.59 to 0.18)	0.29	81	<0.01
Shannon index	-0.11 (-0.27 to 0.05)	0.17	75	<0.01
Simpson index	0.12 (-0.13 to 0.37)	0.34	77	<0.01

SMD, standardised mean difference; CI, confidence interval

Supplementary Table 10. Publication bias assessment by egger's regression test in alpha diversity

**Alpha indexes**	**t**	**df**	***P*-value**
Observed species	-1.21	19	0.24
ACE	0.22	11	0.83
Chao1	0.44	26	0.66
Shannon index	0.47	42	0.64
Simpson index	-0.81	25	0.42

Supplementary Figure 1. Funnel plots of publication bias assessment in alpha diversity

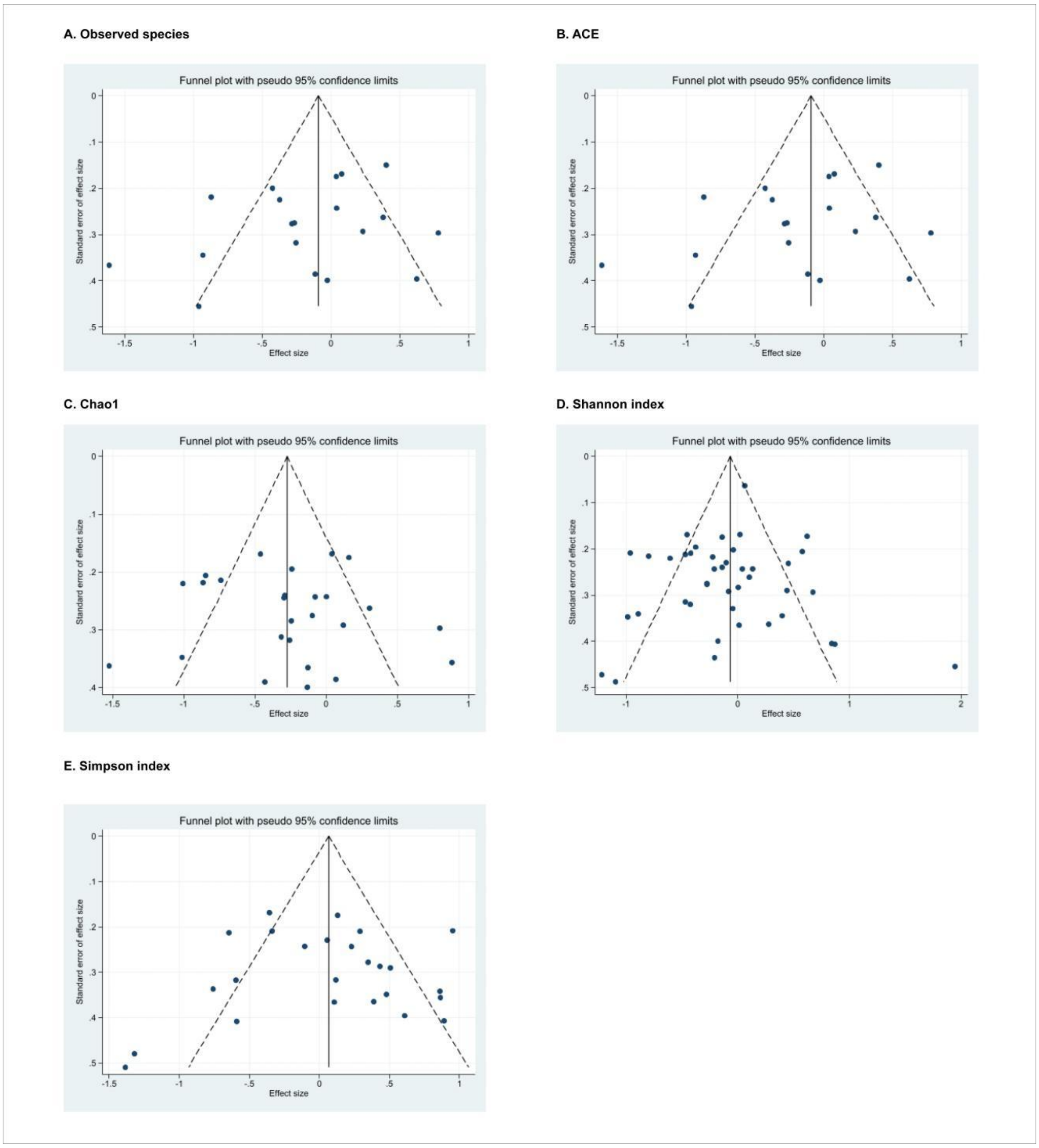

Supplementary Figure 2. Figures for study-level findings of relative abundance of gut microbes

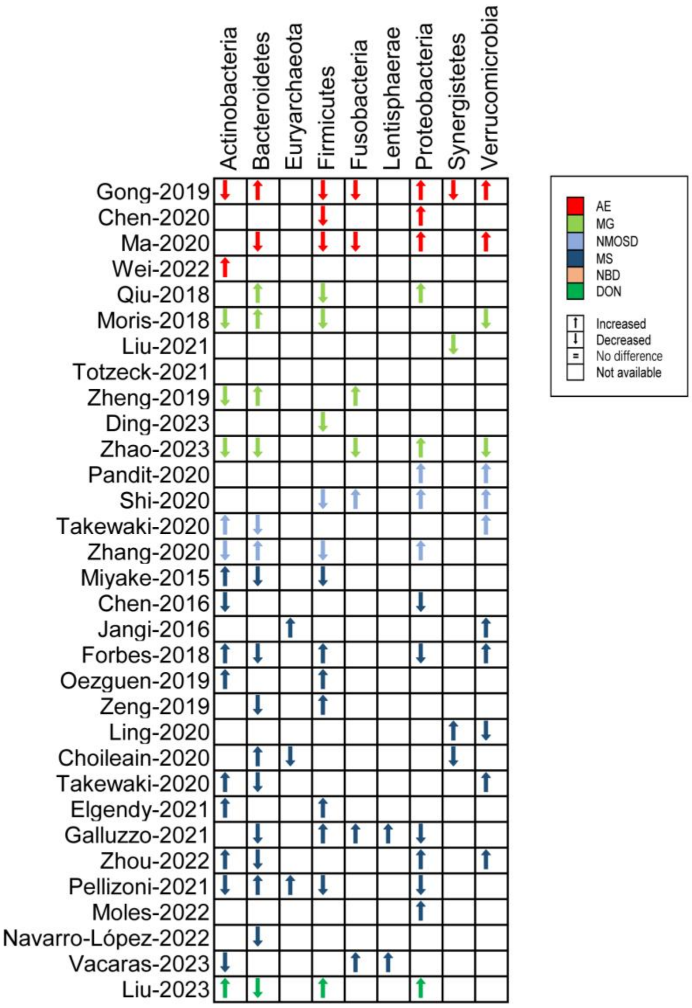

A. Level: phylum

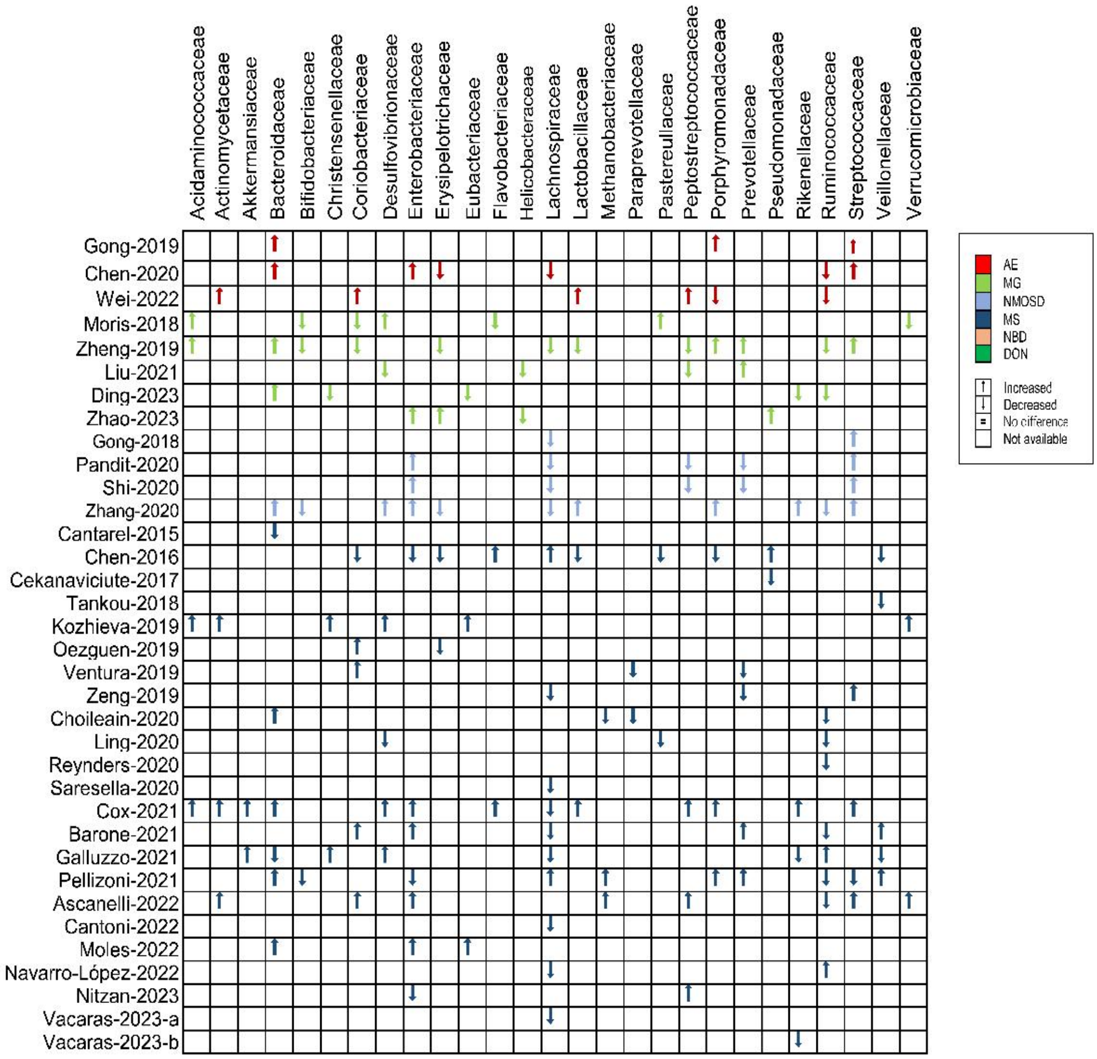

B. Level: family

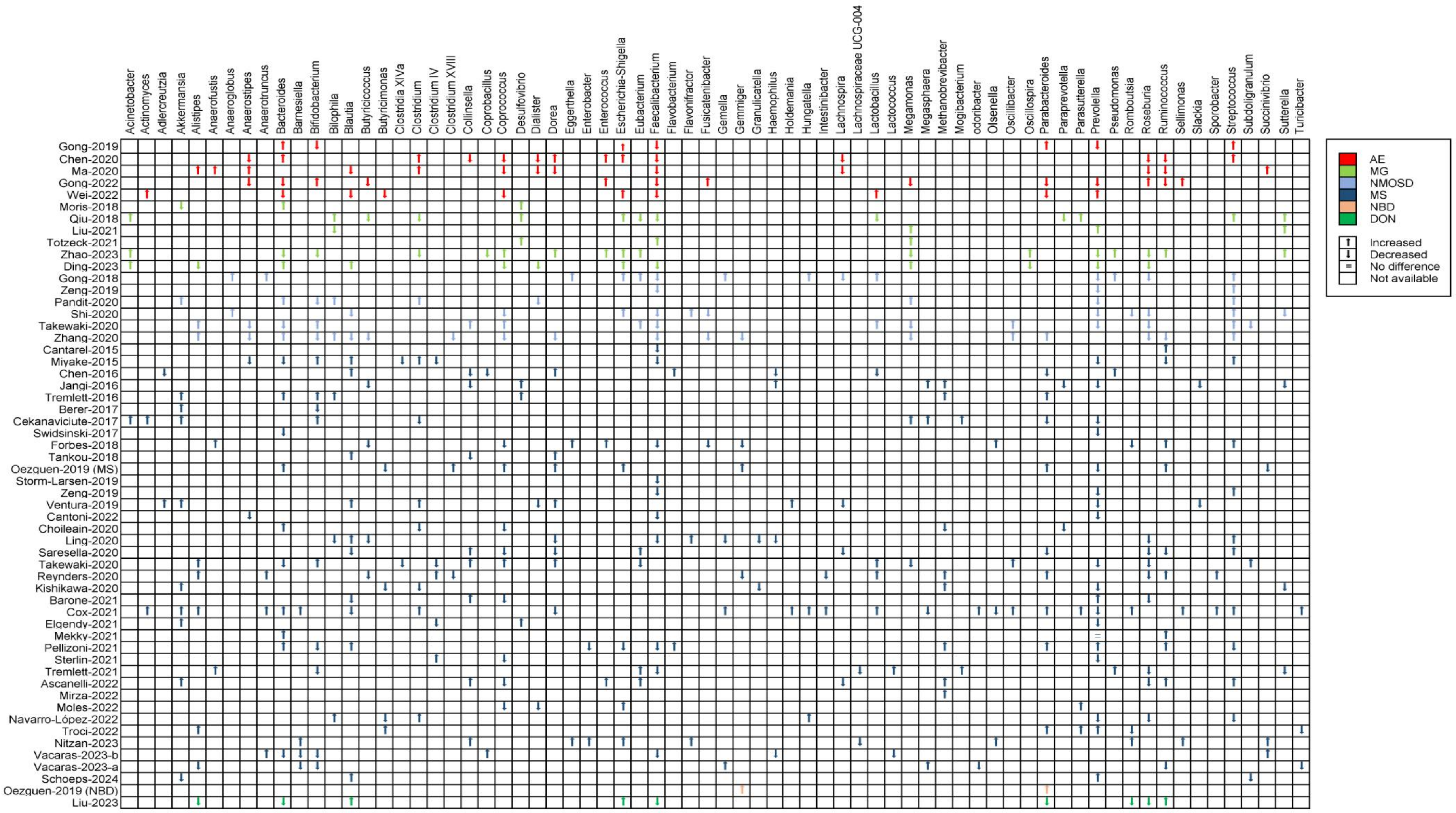

C. Level: genus

Reference

1. Gong X, Liu X, Li C, et al. Alterations in the human gut microbiome in anti-N-methyl-D-aspartate receptor encephalitis. Ann Clin Transl Neurol. 2019;6(9):1771-1781. doi:10.1002/acn3.50874
2. Graus F, Titulaer MJ, Balu R, et al. A clinical approach to diagnosis of autoimmune encephalitis. Lancet Neurol. 2016;15(4):391-404. doi:10.1016/s1474-4422(15)00401-9
3. Herken J, Bang C, Rühlemann MC, et al. Normal gut microbiome in NMDA receptor encephalitis. Neurol Neuroimmunol Neuroinflamm. 2019;6(6)doi:10.1212/nxi.0000000000000632
4. Chen H, Chen Z, Shen L, et al. Fecal microbiota transplantation from patients with autoimmune encephalitis modulates Th17 response and relevant behaviors in mice. Cell Death Discov. 2020;6:75. doi:10.1038/s41420-020-00309-8
5. Gong X, Liu Y, Liu X, et al. Disturbance of Gut Bacteria and Metabolites Are Associated with Disease Severity and Predict Outcome of NMDAR Encephalitis: A Prospective Case-Control Study. FRONTIERS IN IMMUNOLOGY. 2022;12doi:10.3389/fimmu.2021.791780
6. Wei J, Zhang X, Yang F, et al. Gut microbiome changes in anti-N-methyl-D-aspartate receptor encephalitis patients. BMC Neurol. 2022;22(1):276. doi:10.1186/s12883-022-02804-0
7. Ma X, Ma L, Wang Z, et al. Clinical Features and Gut Microbial Alterations in Anti-leucine-rich Glioma-Inactivated 1 Encephalitis-A Pilot Study. Front Neurol. 2020;11:585977. doi:10.3389/fneur.2020.585977
8. Moris G, Arboleya S, Mancabelli L, et al. Fecal microbiota profile in a group of myasthenia gravis patients. Sci Rep. 2018;8(1):14384. doi:10.1038/s41598-018-32700-y
9. Qiu D, Xia Z, Jiao X, Deng J, Zhang L, Li J. Altered Gut Microbiota in Myasthenia Gravis. Front Microbiol. 2018;9:2627. doi:10.3389/fmicb.2018.02627
10. Zheng P, Li Y, Wu J, et al. Perturbed Microbial Ecology in Myasthenia Gravis: Evidence from the Gut Microbiome and Fecal Metabolome. Adv Sci (Weinh). 2019;6(18):1901441. doi:10.1002/advs.201901441
11. Liu P, Jiang Y, Gu S, et al. Metagenome-wide association study of gut microbiome revealed potential microbial marker set for diagnosis of pediatric myasthenia gravis. BMC Med. 2021;19(1):159. doi:10.1186/s12916-021-02034-0
12. Totzeck A, Ramakrishnan E, Schlag M, et al. Gut bacterial microbiota in patients with myasthenia gravis: results from the MYBIOM study. Ther Adv Neurol Disord. 2021;14:17562864211035657. doi:10.1177/17562864211035657
13. Ding XJ, Li HY, Wang H, et al. Altered gut microbiota and metabolites in untreated myasthenia gravis patients. Front Neurol. 2023;14:1248336. doi:10.3389/fneur.2023.1248336
14. Zhao M, Liu L, Liu F, et al. Traditional Chinese medicine improves myasthenia gravis by regulating the symbiotic homeostasis of the intestinal microbiota and host. Frontiers in Microbiology. 2023;13doi:10.3389/fmicb.2022.1082565
15. Cree BA, Spencer CM, Varrin-Doyer M, Baranzini SE, Zamvil SS. Gut microbiome analysis in neuromyelitis optica reveals overabundance of Clostridium perfringens. Ann Neurol. 2016;80(3):443-7. doi:10.1002/ana.24718
16. Gong J, Qiu W, Zeng Q, et al. Lack of short-chain fatty acids and overgrowth of opportunistic pathogens define dysbiosis of neuromyelitis optica spectrum disorders: A Chinese pilot study. Mult Scler. 2019;25(9):1316-1325. doi:10.1177/1352458518790396
17. Wingerchuk DM, Lennon VA, Pittock SJ, Lucchinetti CF, Weinshenker BG. Revised diagnostic criteria for neuromyelitis optica. Neurology. 2006;66(10):1485-9.

doi:10.1212/01.wnl.0000216139.44259.74
18. Zeng Q, Junli G, Liu X, et al. Gut dysbiosis and lack of short chain fatty acids in a Chinese cohort of patients with multiple sclerosis. Neurochemistry International. 2019;129doi:10.1016/j.neuint.2019.104468
19. Pandit L, Cox LM, Malli C, et al. Clostridium bolteae is elevated in neuromyelitis optica spectrum disorder in India and shares sequence similarity with AQP4. Neurol Neuroimmunol Neuroinflamm. 2021;8(1)doi:10.1212/nxi.0000000000000907
20. Shi Z, Qiu Y, Wang J, et al. Dysbiosis of gut microbiota in patients with neuromyelitis optica spectrum disorders: A cross sectional study. J Neuroimmunol. 2020;339:577126. doi:10.1016/j.jneuroim.2019.577126
21. Takewaki D, Suda W, Sato W, et al. Alterations of the gut ecological and functional microenvironment in different stages of multiple sclerosis. Proc Natl Acad Sci U S A. 2020;117(36):22402-22412. doi:10.1073/pnas.2011703117
22. Zhang J, Xu YF, Wu L, Li HF, Wu ZY. Characteristic of gut microbiota in southeastern Chinese patients with neuromyelitis optica spectrum disorders. Mult Scler Relat Disord. 2020;44:102217. doi:10.1016/j.msard.2020.102217
23. Cantarel BL, Waubant E, Chehoud C, et al. Gut microbiota in multiple sclerosis: possible influence of immunomodulators. J Investig Med. 2015;63(5):729-34. doi:10.1097/jim.0000000000000192
24. Miyake S, Kim S, Suda W, et al. Dysbiosis in the Gut Microbiota of Patients with Multiple Sclerosis, with a Striking Depletion of Species Belonging to Clostridia XIVa and IV Clusters. PLoS One. 2015;10(9):e0137429. doi:10.1371/journal.pone.0137429
25. Chen J, Chia N, Kalari KR, et al. Multiple sclerosis patients have a distinct gut microbiota compared to healthy controls. Sci Rep. 2016;6:28484. doi:10.1038/srep28484
26. Jangi S, Gandhi R, Cox LM, et al. Alterations of the human gut microbiome in multiple sclerosis. Nat Commun. 2016;7:12015. doi:10.1038/ncomms12015
27. Tremlett H, Fadrosh DW, Faruqi AA, et al. Gut microbiota in early pediatric multiple sclerosis: a case-control study. Eur J Neurol. 2016;23(8):1308-1321. doi:10.1111/ene.13026
28. Berer K, Gerdes LA, Cekanaviciute E, et al. Gut microbiota from multiple sclerosis patients enables spontaneous autoimmune encephalomyelitis in mice. Proc Natl Acad Sci U S A. 2017;114(40):10719-10724. doi:10.1073/pnas.1711233114
29. Cekanaviciute E, Yoo BB, Runia TF, et al. Gut bacteria from multiple sclerosis patients modulate human T cells and exacerbate symptoms in mouse models. Proceedings of the National Academy of Sciences of the United States of America. 2017;114(40):10713-10718. doi:10.1073/pnas.1711235114
30. Swidsinski A, Doerffel Y, Loening-Baucke V, et al. Reduced Mass and Diversity of the Colonic Microbiome in Patients with Multiple Sclerosis and Their Improvement with Ketogenic Diet. Front Microbiol. 2017;8doi:10.3389/fmicb.2017.01141
31. Forbes JD, Chen CY, Knox NC, et al. A comparative study of the gut microbiota in immune-mediated inflammatory diseases-does a common dysbiosis exist? Microbiome. 2018;6(1):221. doi:10.1186/s40168-018-0603-4
32. Tankou SK, Regev K, Healy BC, et al. A probiotic modulates the microbiome and immunity in multiple sclerosis. Ann Neurol. 2018;83(6):1147-1161. doi:10.1002/ana.25244
33. Kozhieva M, Naumova N, Alikina T, Boyko A, Vlassov V, Kabilov MR. Primary progressive multiple sclerosis in a Russian cohort: relationship with gut bacterial diversity. BMC Microbiol.

2019;19(1):309. doi:10.1186/s12866-019-1685-2
34. Oezguen N, Yalcinkaya N, Kücükali CI, et al. Microbiota stratification identifies disease-specific alterations in neuro-Behçet's disease and multiple sclerosis. Clin Exp Rheumatol. 2019;37 Suppl 121(6):58-66.
35. Storm-Larsen C, Myhr KM, Farbu E, et al. Gut microbiota composition during a 12-week intervention with delayed-release dimethyl fumarate in multiple sclerosis - a pilot trial. Mult Scler J Exp Transl Clin. 2019;5(4):2055217319888767. doi:10.1177/2055217319888767
36. Ventura RE, Iizumi T, Battaglia T, et al. Gut microbiome of treatment-naïve MS patients of different ethnicities early in disease course. Sci Rep. 2019;9(1):16396. doi:10.1038/s41598-019-52894-z
37. Choileáin SN, Kleinewietfeld M, Raddassi K, Hafler DA, Ruff WE, Longbrake EE. CXCR3+ T cells in multiple sclerosis correlate with reduced diversity of the gut microbiome. J Transl Autoimmun. 2020;3:100032. doi:10.1016/j.jtauto.2019.100032
38. Kishikawa T, Ogawa K, Motooka D, et al. A Metagenome-Wide Association Study of Gut Microbiome in Patients With Multiple Sclerosis Revealed Novel Disease Pathology. Front Cell Infect Microbiol. 2020;10:585973. doi:10.3389/fcimb.2020.585973
39. Ling Z, Cheng Y, Yan X, et al. Alterations of the Fecal Microbiota in Chinese Patients With Multiple Sclerosis. Front Immunol. 2020;11:590783. doi:10.3389/fimmu.2020.590783
40. Reynders T, Devolder L, Valles-Colomer M, et al. Gut microbiome variation is associated to Multiple Sclerosis phenotypic subtypes. Ann Clin Transl Neurol. 2020;7(4):406-419. doi:10.1002/acn3.51004
41. Saresella M, Marventano I, Barone M, et al. Alterations in Circulating Fatty Acid Are Associated With Gut Microbiota Dysbiosis and Inflammation in Multiple Sclerosis. Front Immunol. 2020;11:1390. doi:10.3389/fimmu.2020.01390
42. Barone M, Mendozzi L, D'Amico F, et al. Influence of a High-Impact Multidimensional Rehabilitation Program on the Gut Microbiota of Patients with Multiple Sclerosis. Int. J. Mol. Sci. 2021;22(13)doi:10.3390/ijms22137173
43. Cox LM, Maghzi AH, Liu S, et al. Gut Microbiome in Progressive Multiple Sclerosis. Ann Neurol. 2021;89(6):1195-1211. doi:10.1002/ana.26084
44. Elgendy SG, Abd-Elhameed R, Daef E, et al. Gut microbiota in forty cases of egyptian relapsing remitting multiple sclerosis. Iran J Microbiol. 2021;13(5):632-641. doi:10.18502/ijm.v13i5.7428
45. Galluzzo P, Capri FC, Vecchioni L, et al. Comparison of the Intestinal Microbiome of Italian Patients with Multiple Sclerosis and Their Household Relatives. Life (Basel). 2021;11(7)doi:10.3390/life11070620
46. Levi I, Gurevich M, Perlman G, et al. Potential role of indolelactate and butyrate in multiple sclerosis revealed by integrated microbiome-metabolome analysis. Cell Rep Med. 2021;2(4):100246. doi:10.1016/j.xcrm.2021.100246
47. Mekky J, Wani R, Said SM, Ashry M, Ibrahim AE, Ahmed SM. Molecular characterization of the gut microbiome in egyptian patients with remitting relapsing multiple sclerosis. Mult Scler Relat Disord. 2022;57:103354. doi:10.1016/j.msard.2021.103354
48. Mirza AI, Zhu F, Knox N, et al. Metagenomic Analysis of the Pediatric-Onset Multiple Sclerosis Gut Microbiome. Neurology. 2022;98(10):e1050-e1063. doi:10.1212/wnl.0000000000013245
49. Pellizoni FP, Leite AZ, Rodrigues NC, et al. Detection of dysbiosis and increased intestinal permeability in brazilian patients with relapsing–remitting multiple sclerosis. International Journal of

Environmental Research and Public Health. 2021;18(9)doi:10.3390/ijerph18094621
50. Poser CM, Paty DW, Scheinberg L, et al. New diagnostic criteria for multiple sclerosis: guidelines for research protocols. Ann Neurol. 1983;13(3):227-31. doi:10.1002/ana.410130302
51. Sterlin D, Larsen M, Fadlallah J, et al. Perturbed Microbiota/Immune Homeostasis in Multiple Sclerosis. Neurol Neuroimmunol Neuroinflamm. 2021;8(4)doi:10.1212/nxi.0000000000000997
52. Tremlett H, Zhu F, Arnold D, et al. The gut microbiota in pediatric multiple sclerosis and demyelinating syndromes. Ann Clin Transl Neurol. 2021;8(12):2252-2269. doi:10.1002/acn3.51476
53. Ascanelli S, Bombardini C, Chimisso L, et al. Trans-anal irrigation in patients with multiple sclerosis: Efficacy in treating disease-related bowel dysfunctions and impact on the gut microbiota: A monocentric prospective study. Multiple Sclerosis Journal - Experimental, Translational and Clinical. 2022;8(3)doi:10.1177/20552173221109771
54. Bruijstens AL, Molenaar S, Wong YYM, Kraaij R, Neuteboom RF. Gut microbiota analysis in pediatric-onset multiple sclerosis compared to pediatric monophasic demyelinating syndromes and pediatric controls. EUROPEAN JOURNAL OF NEUROLOGY. 2022;doi:10.1111/ene.15594
55. Cantoni C, Lin Q, Dorsett Y, et al. Alterations of host-gut microbiome interactions in multiple sclerosis. EBioMedicine. 2022;76:103798. doi:10.1016/j.ebiom.2021.103798
56. Zhou X,Baumann R, Gao X, et al. Gut microbiome of multiple sclerosis patients and paired household healthy controls reveal associations with disease risk and course. Cell. 2022;185(19):3467-3486.e16. doi:10.1016/j.cell.2022.08.021
57. Moles L, Delgado S, Gorostidi-Aicua M, et al. Microbial dysbiosis and lack of SCFA production in a Spanish cohort of patients with multiple sclerosis. Front Immunol. 2022;13:960761. doi:10.3389/fimmu.2022.960761
58. Navarro-López V, Méndez-Miralles M, Vela-Yebra R, et al. Gut Microbiota as a Potential Predictive Biomarker in Relapsing-Remitting Multiple Sclerosis. Genes (Basel). 2022;13(5)doi:10.3390/genes13050930
59. Troci A, Zimmermann O, Esser D, et al. B-cell-depletion reverses dysbiosis of the microbiome in multiple sclerosis patients. Sci Rep. 2022;12(1):3728. doi:10.1038/s41598-022-07336-8
60. Elsayed NS, Valenzuela RK, Kitchner T, et al. Genetic risk score in multiple sclerosis is associated with unique gut microbiome. Sci Rep. 2023;13(1):16269. doi:10.1038/s41598-023-43217-4
61. Nitzan Z, Staun-Ram E, Volkowich A, Miller A. Multiple Sclerosis-Associated Gut Microbiome in the Israeli Diverse Populations: Associations with Ethnicity, Gender, Disability Status, Vitamin D Levels, and Mediterranean Diet. Int J Mol Sci. 2023;24(19)doi:10.3390/ijms241915024
62. Thirion F, Sellebjerg F, Fan Y, et al. The gut microbiota in multiple sclerosis varies with disease activity. Genome Med. 2023;15(1):1. doi:10.1186/s13073-022-01148-1
63. Vacaras V, Muresanu DF, Buzoianu AD, et al. The role of multiple sclerosis therapies on the dynamic of human gut microbiota. J Neuroimmunol. 2023;378:578087. doi:10.1016/j.jneuroim.2023.578087
64. Schoeps VA, Zhou X, Horton MK, et al. Short-chain fatty acid producers in the gut are associated with pediatric multiple sclerosis onset. Ann Clin Transl Neurol. 2024;11(1):169-184. doi:10.1002/acn3.51944
65. Criteria for diagnosis of Behçet's disease. International Study Group for Behçet's Disease. Lancet. 1990;335(8697):1078-80.
66. Liu Y, Fan H, Shao Y, et al. Gut microbiota dysbiosis associated with different types of demyelinating optic neuritis in patients. Mult Scler Relat Disord..

2023;72doi:10.1016/j.msard.2023.104
